# Multi-resolution perceiver-based residual attention for explainable fault diagnosis in power transmission systems

**DOI:** 10.3389/frai.2026.1903342

**Published:** 2026-09-03

**Authors:** Shantanu Dinesh Wani, S. Abinaya, S. Abirami, R. Priyadarshini

**Affiliations:** School of Computer Science Engineering, Vellore Institute of Technology, Chennai, India

**Keywords:** attention mechanisms, fault diagnosis, multi-task learning, perceiver architecture, spatio-temporal modeling, transmission line faults

## Abstract

Fault diagnosis in power transmission systems requires the simultaneous identification of fault type and location from high-dimensional, multichannel, and temporally evolving measurements. Conventional deep learning approaches may struggle to jointly capture localized transient patterns, global temporal dependencies, and spatially distributed fault signatures while providing interpretable diagnostic information. To address these challenges, a Multi-Resolution Perceiver-Based Residual Attention (MRP-RA) framework is proposed, that combines temporal convolutional feature extraction with a learnable frequency-aware projection branch, Perceiver-based latent modeling, residual spatio-temporal attention, and coupled multi-task learning for fault detection, classification, and localization. The framework processes 256-step windows of 42-channel measurements from a simulated IEEE 5-bus transmission system, where the temporal and frequency-aware representations are fused before being compressed into a latent representation using 32 learnable tokens. Residual temporal and channel attention mechanisms are employed to identify salient temporal regions and feature channels associated with fault signatures, while the multi-task objective jointly optimizes anomaly detection, fault-type classification, and fault localization. Experimental results show that MRP-RA achieves 96.47% fault-type classification accuracy and 97.64% fault-location accuracy across multiple random seeds, while achieving 3.5 × higher inference throughput than the Transformer baseline under the evaluated configuration. These results demonstrate that the proposed combination of multi-resolution feature extraction, latent global modeling, and residual attention can provide accurate joint fault diagnosis with reduced attention-related computational requirements and interpretable attention-based diagnostic insights. The findings support MRP-RA as a promising approach for explainable fault diagnosis in simulated power transmission systems, while further validation across different grid topologies, operating conditions, and real-world measurements is required to establish broader generalizability.

## 1 Introduction

The rapid transition toward renewable energy, along with increasingly uncertain load behavior, is changing the way modern power transmission systems operate. That shift is welcome in one sense because it supports cleaner and more flexible power delivery. However, it also makes monitoring and fault diagnosis significantly more complex. When a fault appears in a tightly connected grid, it can spread quickly, trigger cascading outages, and cause serious economic losses. For that reason, power systems now need diagnostic methods that are accurate, robust, and easy to interpret in real operating conditions (SARMIENTO et al., 2024).

In practice, fault diagnosis is a dual-layered challenge, one has to determine both the fault type and the fault location often at the same time. Grid measurements are high-dimensional, multi-channel, and highly non-stationary, and they are often mixed with noise from switching events, operating fluctuations, or the environment itself. An effective model must be able to look at brief transients that represent fault initiation and at the same time provides a long run perspective of the sequence. In safety critical systems, it is necessary to know precisely when, and where, a fault began in a system, so that action can be taken decisively (Lalouani, 2025).

Although the field of deep learning advances, there are several issues that continue to rise in fault diagnosis. It is not easy to do long-sequence modeling. Convolutional neural networks (CNNs) excel at detecting local patterns, yet tend to overlook bigger global patterns. Recurrent models such as LSTMs follow sequences naturally and can handle sequences of hundreds of steps. However, they lack the global context mechanisms that allow simultaneous multi-scale reasoning across the full diagnostic window (Belagoune et al., 2021). Transformer-based techniques model long-range interactions effectively but incur quadratic computational cost with sequence length. Even at the moderate *T* = 256 window used in this work where each time step carries 42 synchronized sensor channels where the full self-attention mechanism requires O(*T*^2^) = 65, 536 pairwise computations, making it measurably costlier than the Perceiver bottleneck we propose [O(*T*·*L*) = 8, 192 at *L* = 32], a difference confirmed empirically in Section 5.2.

It is also common in the literature to over-emphasize the time domain. That is as far as it goes, it omits spectral behavior and harmonic changes which tend to be highly informative in cases of fault. The other weakness is that the classification of fault types and fault location are often considered to be independent activities despite inherent correlations between the two. On top of that, many existing systems still behave like “black boxes,” or they produce attention maps that are difficult to connect to actual physical behavior in the grid. Even the training side of the problem, including optimization choices and convergence stability, is sometimes handled a little too casually for a task this sensitive.

To work around these problems, the Multi-Resolution Perceiver-based Residual Attention model is proposed. The model combines temporal convolutional encoding with a genuine FFT-based spectral branch: ‘torch.fft.rfft' is applied over the time dimension of each input window, the magnitude spectrum is extracted, and a learned linear projection maps these spectral features into the shared latent space which enables the model to capture harmonic distortions and spectral energy redistributions that are diagnostically distinct from the time-domain waveform representation. The multi-resolution design is convenient since fault signatures do not often exist in a single domain. To process long sequences more effectively, a Perceiver-based latent attention module is used. The model can decrease the computational cost of full self-attention [O(*T*^2^)] by mapping the input into a latent space [O(*T*·*L*)] but retains the global context required to make a diagnosis. That is, it does not suffer some of the traditionally scaling issues, without compromising the larger signal structure.

Residual temporal and channel attention modules are also added to emphasize the signal components which is the most significant to the fault. They render model more interpretative, as they are able to depict where the disturbance originates, and the extent to which it is spread throughout the transmission lines. On top of the architecture, being somewhat more critical and critical because the issue of training stability is of concern when the data are disproportionate and the task at hand is a question of life-and-death. The suggested method has better performance on the IEEE 5-Bus Transmission Line Fault (TL Fault) dataset (Custodio et al., 2023) as compared to standard CNN, LSTM, and transformer-based baselines.

The main contributions of this study are as follows:

- **Unified multi-resolution fault diagnosis system:** This is proposed to be an entire system comprised of a temporal convolutional encoder with a true FFT-based branch of the spectral feature, allowing concurrent time transient and harmonic spectral signatures capture. An ablation study (Section 5.6) confirms that every component makes a significant contribution to the performance.
- **Scalable latent attention with Perceiver:** A perceiver-based model is presented for the power fault. The diagnosis problem with the support for scalable long-sequence processing, O(*T*·*L*) computation. While retaining all the global information.
- **Residual spatio-temporal attention for interpretability:** A residual attention framework is developed to decompose interpretability into temporal and channel-wise outputs, providing physically interpretable insights into fault initiation and propagation.
- **Coupled multi-task learning:** Fault diagnosis is formulated as a coupled learning task involving anomaly detection, fault classification, and fault localization, enabling the learning of shared representations beneficial across tasks.
- **Systematic analysis of training dynamics:** Optimization and convergence dynamics are systematically analyzed, including a full ablation study (Section 5.6) isolating the contribution of each architectural component, a focal-loss γ sweep, and a Perceiver latent-count sweep, to improve model stability and generalization on unbalanced fault datasets.

The remaining part of this study is organized as follows. In section II, the related work is reviewed. Section III presents the proposed methodology. Section IV outlines the experimental setup. In Section V, the experimental results and analysis of interpretability are discussed. Section VI concludes the study.

## 2 Related works

Fault detection, classification, and localization techniques in power systems have gone through a huge evolution with the development of machine learning and deep learning. This is that continuation and hence the shift of the field toward the new paradigms of attention and latent modeling is observed.

### 2.1 Traditional machine learning approaches

The initial diagnostic systems mostly relied on manually constructed features along with classical machine learning. Extreme Learning Machines (ELM) was commonly used due to their low cost of computing and fault classification speed (Goni et al., 2023). Similarly, machine learning has been used together with frequency response analysis to identify faults in transformers through the spectral particularity of signals (Maseko et al., 2025).

With the increase in data dimensionality, deep autoencoders were proposed to facilitate the unsupervised learning of features on high data dimensions of industrial data (Kordon, 2023). It was also during this period that hybrid feature extraction algorithms, combining signal processing and machine learning, were developed to solve combined classification and localization problems (Chen et al., 2017). More recently, graph-based inductive methods such as GraphSAGE have improved localization by explicitly modeling the relationships between different grid components (Wang and Hu, 2025). Additionally, contrastive representation learning has been explored to help models remain robust under changing operating conditions (Zhang et al., 2023). Contrastive prototype learning has also been applied specifically to class-imbalanced fault scenarios in EV charging infrastructure, demonstrating that prototype-driven objectives can substantially recover minority-class detection performance without requiring architectural overhaul (Lei et al., 2026).

### 2.2 Deep learning and spatio-temporal modeling

Deep learning has essentially enhanced fault diagnosis in the sense that it enables models to learn hierarchical representations directly on raw data. The CNN- and LSTM-based models have been highly implemented to learn the spatial and time dependencies of transmission line faults (Belagoune et al., 2021). Hybrid CNN-LSTM models go a step further to enhance this performance by integrating local feature extraction with long-range sequential tracking Custodio et al., (2024). Notably, (Custodio et al. 2024) evaluate their federated 1D-CNN-LSTM model on the same publicly available IEEE 5-bus transmission-line fault dataset (Custodio et al., 2023) adopted in this study; whereas their work targets a federated-learning deployment setting, the present work introduces a distinct architecture combining dual-domain feature extraction, Perceiver-based latent attention, and residual spatio-temporal attention within a multi-task learning framework which was evaluated under an independent train/validation/test protocol, distinguishing our contribution from this prior use of the same underlying dataset.

To better generalize to unseen or rare fault conditions, researchers have recently investigated contrastive learning frames in time series specifically (Rombach et al., 2021; Liu et al., 2025). These trends are also manifested in detailed surveys which indicate a definite industry-wide shift toward deep learning to improve grid resilience (SARMIENTO et al., 2024).

### 2.3 Graph-based and spatio-temporal modeling

Graph neural networks (GNNs) were proposed to represent the physical structure of power grids to model the spatial dependencies between networks even more effectively (Rimkus et al., 2025). These have since been generalized to spatio-temporal models, including graph attention networks with LSTMs to detect machinery faults (Singh et al., 2024). Variational graph attention autoencoders to monitor complex industrial processes (Lv et al., 2023) and graph-based learning to detect the spatio-temporal parameters in power grids (Yuan et al., 2025) are other innovations.

### 2.4 Attention-based and interpretable models

One of the pillars that has been developed to help improve accuracy and transparency in models is attention mechanisms. The application of spatio-temporal attention has been successfully applied to forecasting smart grids and in other activities related to faults (Cavus and Allahham, 2025). In addition, they are training more interpretable energy forecasting models by using attention-based fusion approaches (Li and Chen, 2025). Physics-inspired time-frequency feature extraction combined with lightweight neural networks has also shown strong performance for power quality disturbance classification, providing a direct precedent for the dual-domain (time + frequency) approach adopted in this work (Hou et al., 2025). Notably, the Explainable AI (XAI) models that implement attention currently provide the insights into the model-decisions that are highly required and that can be implemented in safety-critical power systems (Ghahfarokhi et al., 2025; Lalouani, 2025).

### 2.5 Latent modeling of perceiver

Perceiver-based architectures are a recent, powerful and scalable solution to high-dimensional temporal data. These models are able to achieve global dependencies far more effectively than standard transformers using latent arrays and cross-attention. Perceiver IO has been proven to be able to compress signals already with strong memory limits (Lebonvallet et al., 2024), and analogous latent model approaches are already proving helpful to time-series prediction (Le et al., 2023).

### 2.6 Research gap and positioning

Despite all these improvements, there are still numerous significant limitations:

- Coherent frameworks, integrating fruitfully time and frequency domain representations, are lacking.
- Most of the models are poor with regard to multi-resolution time scale properties.
- Graph-based and attention-based models are also usually computationally expensive.
- Physical properties of deep learning methods are still unsatisfactory (in terms of decent interpretability).

The Multi-Resolution Perceiver with Residual Attention (MRP-RA) framework proposed is specifically created to cover these gaps. The integration of multi-resolution extracting, dual-domain learning, and Perceiver-style latent models to a multi-task setting provides a solution to the worth of the best result and the reliable interpretability.

## 3 Multi-resolution perceiver-based residual attention framework

To overcome the multi-task difficulties of power transmission analysis, Multi-Resolution Perceiver-Based Residual Attention (MRP-RA) framework is formulated. This architecture is specifically to serve high dimensional inference problems in multi-channel grid data, fault events are complicated spatio-temporal transients, which are often difficult to distinguish background noise.

### 3.1 Overall multi-task explainable framework

**Figure 1:**
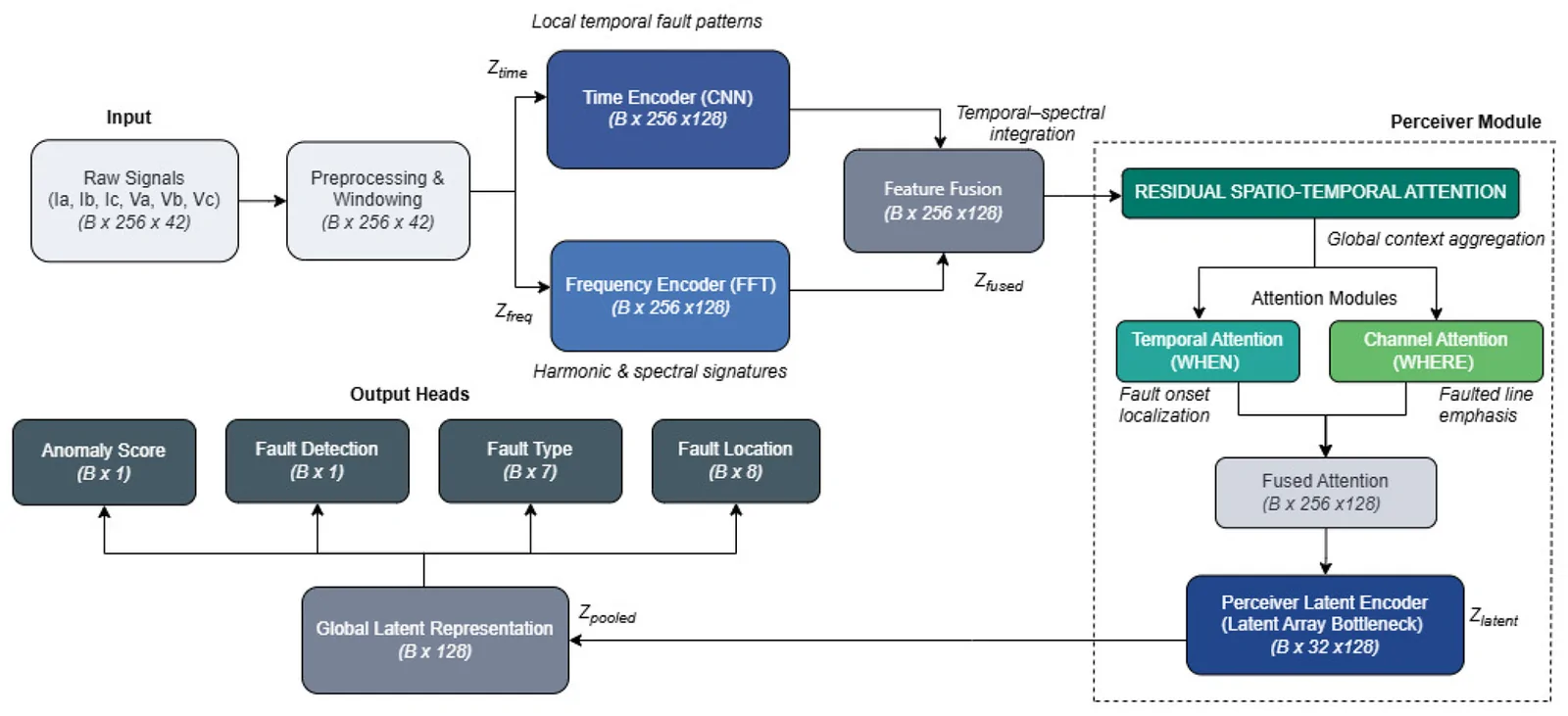
Proposed multi-task architecture. Raw signals are processed via dual streams (time and frequency encoders) and fused using a Perceiver Latent Encoder to generate anomaly scores, fault classifications, and locations.

To achieve a balance between scalability and interpretability, the MRP-RA architecture features a Perceiver-based latent bottleneck that processes the different features of the input domain, along with feature extraction in both domains. In accordance with the discussion in Figure 1, the process of diagnosis is divided into three strategic steps:

1. **Dual-stream extraction**: The raw multi-channel signals are encoded parallel, one by Time (CNN) and the other by Frequency (FFT style). This guarantees that the model is able to capture immediate distortions on the waveform and the underlying harmonic patterns that characterize a particular type of fault.

2. **Global latent learning**: These high-dimensional representations are learned as a small, consolidated latent embedding with the Perceiver module, which performs its computations as a computational “filter” on global context.

3. **Multi-task decoding**: The model utilizes this reduced context to encourage four parallel output heads to generate anomaly scores, classifications, and location estimates, with special attention modules included to provide the “when” and “where” information required by human-in-the-loop verification.

The structure provides some computational efficiency and expressiveness not afforded by most traditional modular pipelines by the decoupling of local-feature extraction and global reasoning.

#### 3.1.1 Temporal convolutional encoder

The temporal encoder will use a sequence of one-dimensional (1D) convolutional layers to the input sequence to extract hierarchical features. The input tensor is given by the *Z*^(0)^ = *X* ∈ ℝ^*B*×*T*×*C*^, where *B* is the batch size, *T* is the time length, and *C* is the number of feature channels. The operation of each convolutional layer is to learn the “shape” of fault transients as follows:


$$\begin{array}{l} Z^{\left(l\right)}=\sigma \left(W^{\left(l\right)}*Z^{\left(l-1\right)}+b^{\left(l\right)}\right),\;l=1,2,3 \end{array}$$ (1)

In Equation 1, 1D convolution in the time domain is denoted by, where *W*^(*l*)^ and *b*^(*l*)^ are the learnable parameters, and σ(·) is the ReLU activation function. To have a closer look at one of the channels, the operation can be written as


$$\begin{array}{l} {\left(Z^{\left(l\right)}\right)}_{t,k}=\sigma \left(\overset{K}{\underset{i=1}{\sum }}\overset{C_{l-1}}{\underset{c=1}{\sum }}W_{i,c,k}^{\left(l\right)}\cdot Z_{t+i,c}^{\left(l-1\right)}+b_{k}^{\left(l\right)}\right) \end{array}$$ (2)

In Equation 2, *K* = 5 is the kernel size (with stride = 1, padding = 2 for same-padding that preserves the *T* = 256 temporal dimension throughout, and dilation = 1), and k indexes the output channels. The channels progress as 42 → 64 → 96 → 128 across the three convolutional layers, each followed by BatchNorm1d and GELU activation. This design is deliberately chosen to enable the model to learn multi-scale patterns. Whereas early layers identify the localized disturbances, deeper layers encode the more global temporal abstractions that are used to define fault propagation. The receptive field, computed as *R* = *n*_*layers*_(*K*−1)+1 = 3(5 − 1)+1 = 13 time steps, encompasses the approximately 5-step fault-transient window identified in the temporal attention analysis (Section 5.5.1), confirming that the encoder captures fault-onset patterns within a single pass through all three layers without requiring dilated convolutions.


$$\begin{array}{l} Z_{time}=Z^{\left(3\right)}\in {\mathbb{R}}^{B\times T\times D} \end{array}$$ (3)

Using *Z*_*time*_ of Equation 3 to represent the last feature map of this branch.

#### 3.1.2 Spectral feature extraction via FFT

Faults in power grids redistribute spectral energy across frequency bands and introduce harmonic distortions that are diagnostically distinct from time-domain waveform shape. To capture these characteristics, the frequency branch computes a genuine real-valued FFT magnitude spectrum from the input signal.

For an input window *X* ∈ ℝ^*B*×*T*×*C*^, the real-valued FFT is applied over the time dimension:


$$\begin{array}{l} M_{freq}=|FFT_{T}\left(X\right)|\in R^{B\times \left(\frac{T}{2}+1\right)\times C} \end{array}$$ (4)

In Equation 4, *FFT*_*T*_(·) denotes torch.fft.rfft applied along dimension T and |·| extracts the magnitude spectrum. The resulting magnitude features capture the per-channel distribution of spectral energy across T/2+1 frequency bins. These are flattened and projected into the shared D-dimensional latent space:


$$\begin{array}{l} Z_{freq}=\mathrm{Flatten}\left(M_{freq}\right)W_{freq}+b_{freq}\in R^{B\times D} \end{array}$$ (5)

$W_{\text{freq}}\in {\mathbb{R}}^{C\left(\frac{T}{2}+1\right)\times D}$ is a learned projection matrix in Equation 5. This branch accumulates the data over the entire time period and can be used to represent more complex energy patterns (and harmonics) that are typical for certain fault types not captured by a per-timestep linear projection. *Z*_*freq*_ is then sent out again in the *T* time dimension and added together at the end of *Z*_*time*_ for fusion.

#### 3.1.3 Feature fusion

The optimization of temporal stream (*Z*_*time*_) and frequency stream (*Z*_*freq*_) of Equation 6 are merged in an effort to get a global picture of the grid. Initially concatenate them in the channel direction:


$$\begin{array}{l} Z_{concat}=\left[Z_{time};Z_{freq}\right]\in {\mathbb{R}}^{B\times T\times 2D} \end{array}$$ (6)

A learning fusion mechanism is used to learn to fuse these heterogeneous representations:


$$\begin{array}{l} Z_{fused}=\sigma \left(W_{fusion}Z_{concat}+b_{fusion}\right) \end{array}$$ (7)

This operation (Equation 7) sees that the downstream Perceiver module gets a rich information signal that explains both local dynamics and global spectral trends.

#### 3.1.4 Perceiver-based latent encoding

The main component of this architecture is Perceiver, a learnable array of latents $L_{0}\in {\mathbb{R}}^{B\times 32\times 128}$ that does not exhibit the scalability issues of conventional transformers. It works in a three-step cycle which takes the original latent query and converts it into a context-sensitive global representation:

1. **Cross-attention**: The module “queries” the high dimensional *Z*_fused_ input and aggregates the most relevant information into the compact latent tokens:

$$\begin{array}{l} Z^{\left(1\right)}=\text{LayerNorm}\left(L_{0}+\text{CrossAttn}\left(L_{0},Z_{\text{fused}}\right)\right) \end{array}$$ (8)

2. **Latent self-attention**: The model explores worldwide connections between these latent vectors to learn about association between different elements of the fault sequence:

$$\begin{array}{l} Z^{\left(2\right)}=\text{LayerNorm}\left(Z^{\left(1\right)}+\text{SelfAttn}\left(Z^{\left(1\right)}\right)\right) \end{array}$$ (9)

3. **Feedforward network (FFN)**: Finally, a position-wise FFN increases the ability of these representations with non-linear transformations:

$$\begin{array}{l} Z^{\left(3\right)}=\text{LayerNorm}\left(Z^{\left(2\right)}+\text{FFN}\left(Z^{\left(2\right)}\right)\right) \end{array}$$ (10)

Final value of this refinement cycle with respect to the final value of this refinement cycle in terms of the final value of previous Equations 8–10 is


$$\begin{array}{l} Z_{\text{latent}}=Z^{\left(3\right)}\in {\mathbb{R}}^{B\times L\times D} \end{array}$$ (11)

The depth of the reasoning layers and length of the input signal are effectively decoupled by compressing the sequence to a fixed-size latent space (*L* = 32) as illustrated in Equation 11. This architectural choice reduces the computational complexity of the latent-attention component from O(*T*^2^) to O(*T*·*L*), a reduction expected to lower memory and compute requirements relative to full self-attention, particularly at longer sequence lengths; confirming this benefit on constrained industrial edge hardware would require platform-specific latency and memory benchmarking beyond the scope of the present study.

#### 3.1.5 Global pooling

To get the data ready to make final prediction, the latent representation $Z_{\text{latent}}\in {\mathbb{R}}^{B\times L\times D}$ is taken and global mean pooling:


$$\begin{array}{l} Z_{\text{pooled}}=\frac{1}{L}\overset{L}{\underset{i=1}{\sum }}Z_{\text{latent},i} \end{array}$$ (12)

The reason why mean pooling is selected because it is inherently stable as demonstrated in Equation 12. It aggregates context across all 32 tokens of the latent and provides a powerful global signal of the event.

#### 3.1.6 Multi-task output heads

It is then fed into four parallel fully connected heads which reflect the multi-task nature of the problem:

a. **Fault detection**: A binary value to give immediate alert.
b. **Anomaly detection**: A continuous health-monitoring score.
c. **Fault type classification**: Classifying the signal into one of seven fault classes.
d. **Fault localization**: Localizing the event on eight possible locations.

#### 3.1.7 Architectural insights

**Figure 2:**
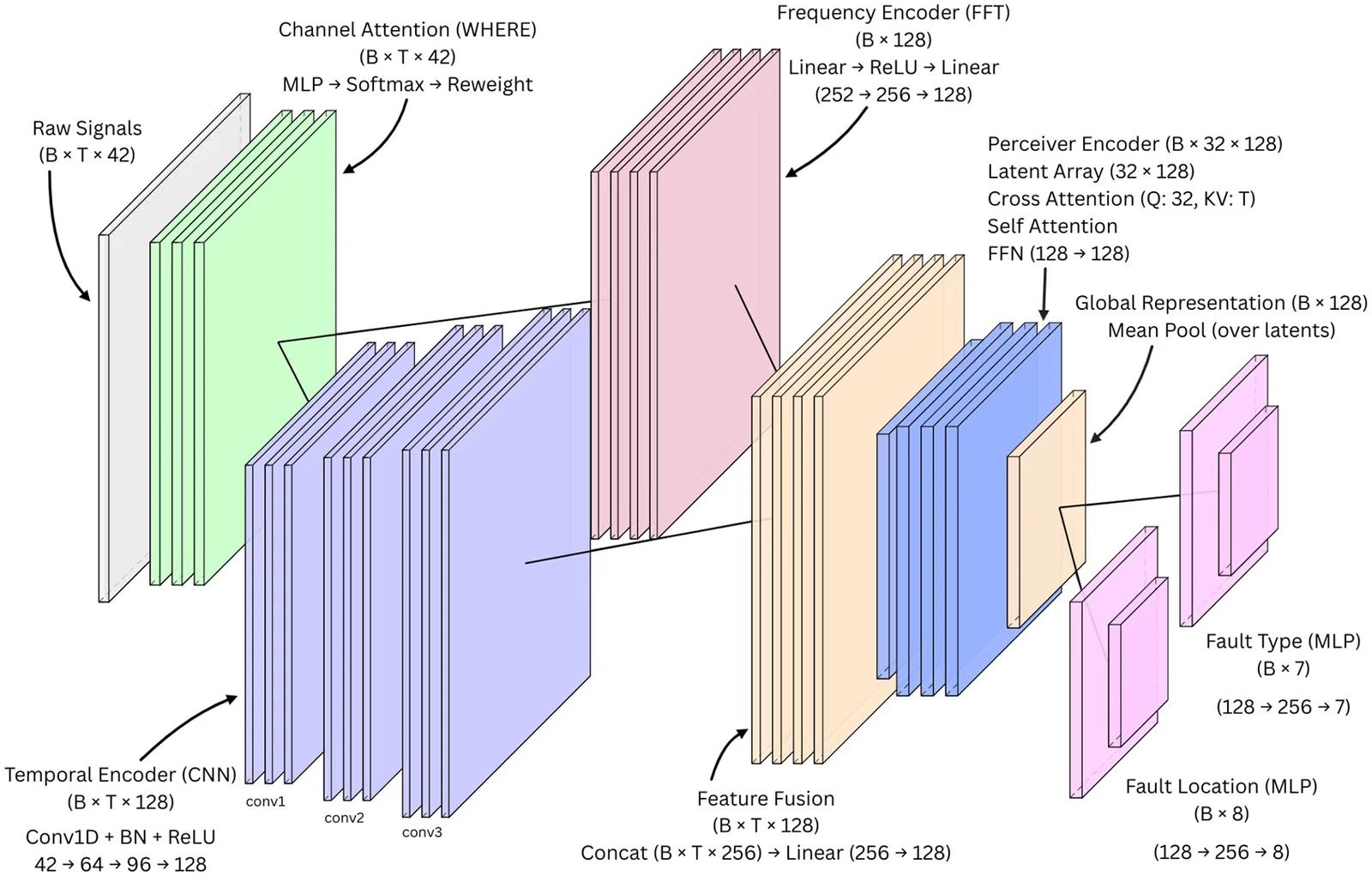
Detailed architecture of the proposed MRP-RA framework. The model processes raw multi-channel temporal signals through a temporal convolutional encoder and a frequency projection branch, followed by feature fusion, Perceiver-based latent encoding, attention mechanisms, and multi-task prediction heads.

As seen in Figure 2, MRP-RA framework offers three unique benefits as compared to existing approaches. To start with, its multi-resolution approach will ensure that no time or frequency diagnostic information is wasted. Second, its Perceiver-based latent bottleneck enables the model to be highly scaled, which enables long sequences to be fed through the model, resulting in crashes of regular Transformer models. Finally, as a multi-task framework, the framework establishes a “synergy” in the training process; as the model is trained to tackle classification and localization jointly, the model builds more robust and generalized feature representations. The outcome, under the simulated IEEE 5-bus conditions evaluated in this study, is a system that is both accurate and computationally efficient relative to the transformer baseline; extending these results to real-world grid deployment would require validation on field data, which we identify as an important direction for future work.

### 3.2 Data representation and preprocessing

A multi-channel time-dependent input tensor *X* ∈ ℝ^*B*×*T*×*C*^ that is run through this framework and *B* is the batch size and *T* = 256 is the constant window size (time steps) is the input. The *C* = 42 synchronized measurement channels in the input give us a detailed picture of the situation in the grid in terms of three phase currents (*I*_*a*_, *I*_*b*_, *I*_*c*_) and voltages (*V*_*a*_, *V*_*b*_, *V*_*c*_) measured on various transmission lines.

Due to the two large-scale quantities that the model is trying to solve for, voltage (kV) and current (kA), the raw signals provided can be a cause of discrepancies in scale difference, which may lead to biases in the learning process toward larger scale characteristics. To equalize the playing field, the Z-score normalization of each channel is used with statistics based purely on the training set:


$$\begin{array}{l} x_{t,c}^{\prime }=\frac{x_{t,c}-\mu_{c}}{\sigma_{c}} \end{array}$$ (13)

In the Equation 13, μ_*c*_ and σ_*c*_ are the average and the standard deviation of channel *c*. This per-channel normalization makes the process of the feature extraction stable and not to give the domination of a certain type of measurement on the latent representation where *X*′ ∈ ℝ^*B*×*T*×*C*^ denotes the normalized input tensor.

**Figure 3:**
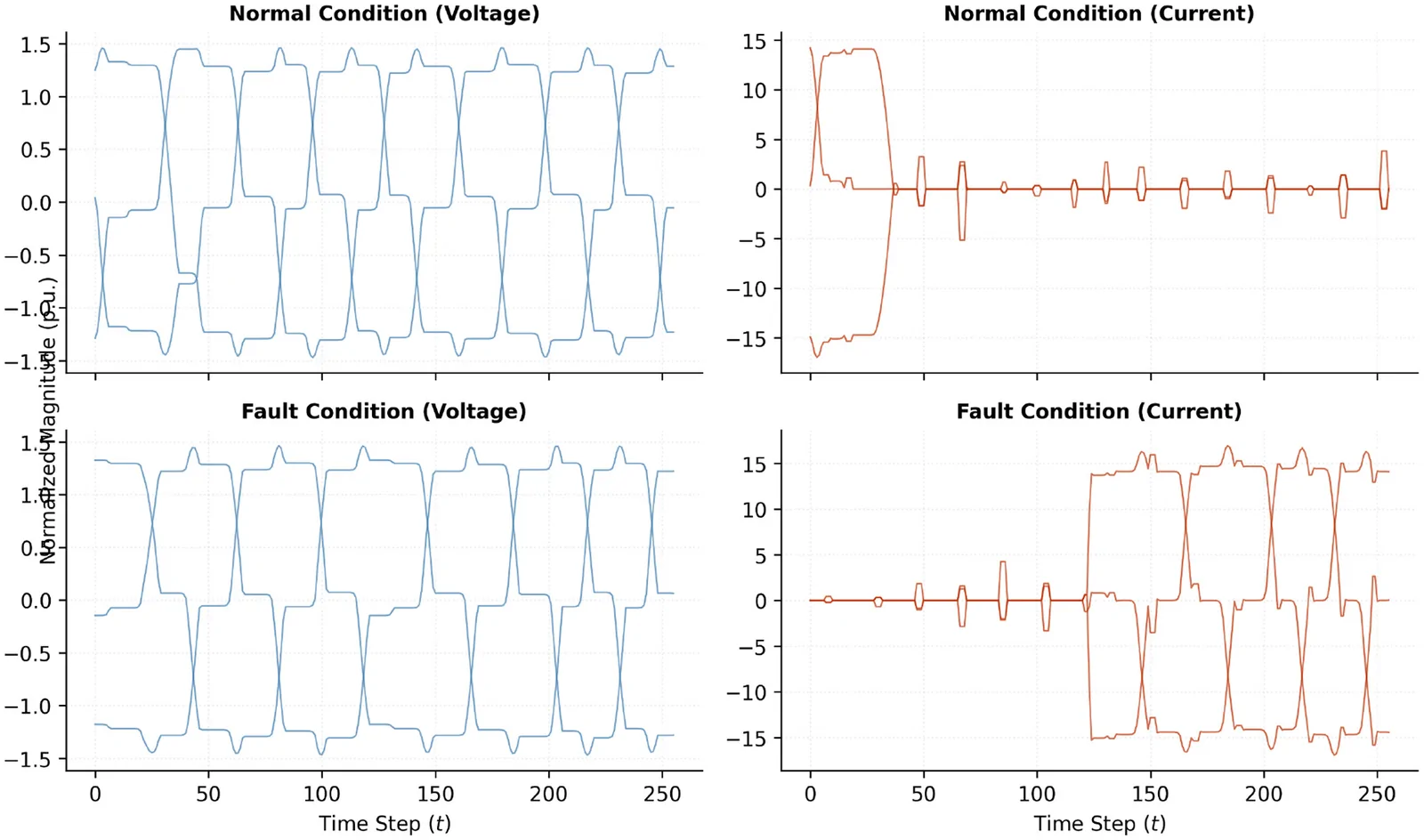
Comparison of normal and fault conditions. Fault scenarios exhibit voltage distortions and significant current deviations across phases.

Transmission line faults are physically manifested by sharp discontinuities of the steady-state sine waves of the grid. Such events may be characterized by distinct signatures, and this model is biased to detect these events as illustrated in Figure 3:

- **Voltage sags:** When a fault occurs, the initial initiation of the fault normally leads to the sudden decrease of the magnitude of voltage on the faulted phases.
- **Current surges:** At the same time, the model is supposed to respond to high current spikes that are normally quite high in comparison with the operating levels.
- **Harmonic distortion:** Faults redistribute spectral energy, creating high frequency components that are important fault-type discrimination cues, in addition to magnitude shift as a simple magnitude attribute.

**Figure 4:**
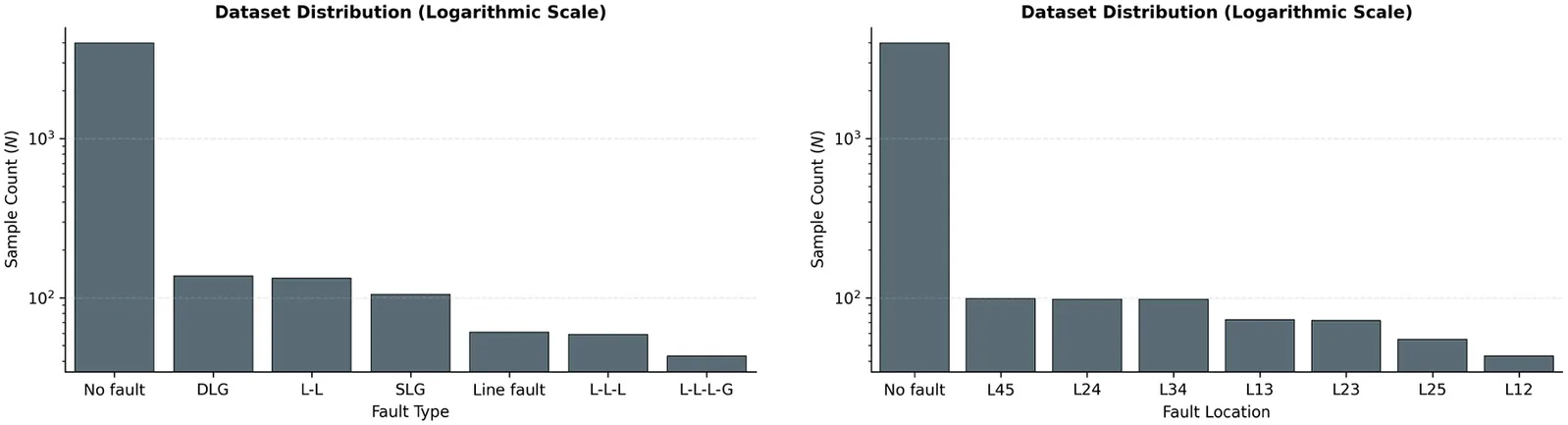
Combined visualization of fault type distribution **(left)** and fault location distribution **(right)**.

In addition to the intricacy of the signals, the dataset poses a considerable needle in a haystack problem as the classes are heavily unbalanced. Figure 4 shows that most of the samples in the samples fall in the Overwhelming Majority category that consists of over 85% “No Fault.” Rare, but dangerous types of fault include L-L-L or L-L-L-G, which are highly under-represented. Similarly, the localization task is further complicated by the fact that the distribution of the faults along the lines is uneven. These properties require a model that is capable of learning highly discriminative features with only a small number of examples.

A **Channel-wise attention reweighting** mechanism is also used to refine this input further, before the temporal encoding takes place. This is aimed at enabling the model to “pre-filter” the incoming data to dynamically highlight informative information and downplay measurement noise or redundant data.

The computation of a channel attention vector β ∈ ℝ^*B*×1 × *C*^ is as follows:


$$\begin{array}{l} \beta =\sigma \left(f_{\theta }\left({\text{Pool}}_{T}\left(X^{\prime }\right)\right)\right) \end{array}$$ (14)

In Equation 14, Pool_*T*_(·) Pool achieves mean pooling in the time dimension to obtain a global “snapshot” of the activity in each channel, and *f*_θ_(·) is a small training of MLP that learns the relative weight of those channels. The sigmoid activation σ(·) makes the weights lie in the range [0, 1]. It is then reweighted through element-wise scaling:


$$\begin{array}{l} X^{\text{attn}}=\beta ⊙X^{\prime } \end{array}$$ (15)

This initial focus in the form of the Equation 15 is a gatekeeper which makes the model consider the most physically relevant phases and transmission lines before it sinks into the convoluted time analysis. This serves to minimize noise propagation and enhances the model to resist non-relevant variations in the multi-channel stream. Note that the global temporal pooling in Equation 14 serves strictly to compute a macro channel-reweighting vector **w** ∈ ℝ^*C*^ based on total windowed transient energy across sensor phases. This operation does not reduce the temporal dimension; the full, uncompressed sequence *X* ∈ ℝ^*C*×*T*^ is preserved and multiplied by **w**, ensuring that short-duration, high-frequency transient signatures remain entirely intact for fine-grained temporal modeling in subsequent encoder layers.

### 3.3 Multi-resolution feature extraction

A dual-stream feature extraction strategy is used to capture as much of the subtle interaction of both the temporal and spectral properties of fault signals as possible. This title is assigned to ensure that the model can also follow the immediate physical variations of a waveform, as well as the larger spectral variations which are so typical of some faults.

#### 3.3.1 Temporal feature extraction

This convolutional encoder operates on the normalized input sequence, and targets localized temporal patterns:


$$\begin{array}{l} Z_{time}\in {\mathbb{R}}^{B\times T\times D} \end{array}$$ (16)

Such a representation in Equation 16 is intended to capture the “sharp” aspects of a fault such as transient disturbances, sudden distortions of the waveforms, and the temporal dependencies which develop instantly a fault takes place.

#### 3.3.2 Frequency-aware feature extraction

The frequency encoder computes the FFT magnitude spectrum and projects it into the shared D-dimensional space, as described in Section 3.1.2 (Equations 4, 5). The resulting representation $Z_{freq}\in {\mathbb{R}}^{B\times D}$ captures harmonic and spectral energy content integrated across the full T-step window.

#### 3.3.3 Representation alignment

To make these two different views compatible, both the frequency and the temporal representations are put into the same dimensional space:


$$\begin{array}{l} Z_{time},Z_{freq}\in {\mathbb{R}}^{B\times T\times D} \end{array}$$ (17)

It is a significant architectural step in this coordinate (Equation 17). It ensures mathematical consistency to downstream fusion without undergoing a complicated broadcasting or data replication. The temporal encoder viewpoint of the locale and the frequency-conscious projection viewpoint of the globe create a multi-resolution basis which is particularly preferred to the challenges of fault diagnosis.

### 3.4 Perceiver-based global latent encoding

Long-range dependencies of high dimensional multi-channel time-series data are both a large computational burden to capture faulty behaviors and to diagnose them. Simple models of self-attention scale quadratically with sequence length [O(*T*^2^)] and thus are less efficient at larger time window size (*T* = 256) or sensor density. Moreover, since multi-channel grid signals such as synchronized three-phase voltages and currents can be tightly linked, the direct application of self-attention to raw sequences can often involve redundant computations of repetitive time structures.

**Figure 5:**
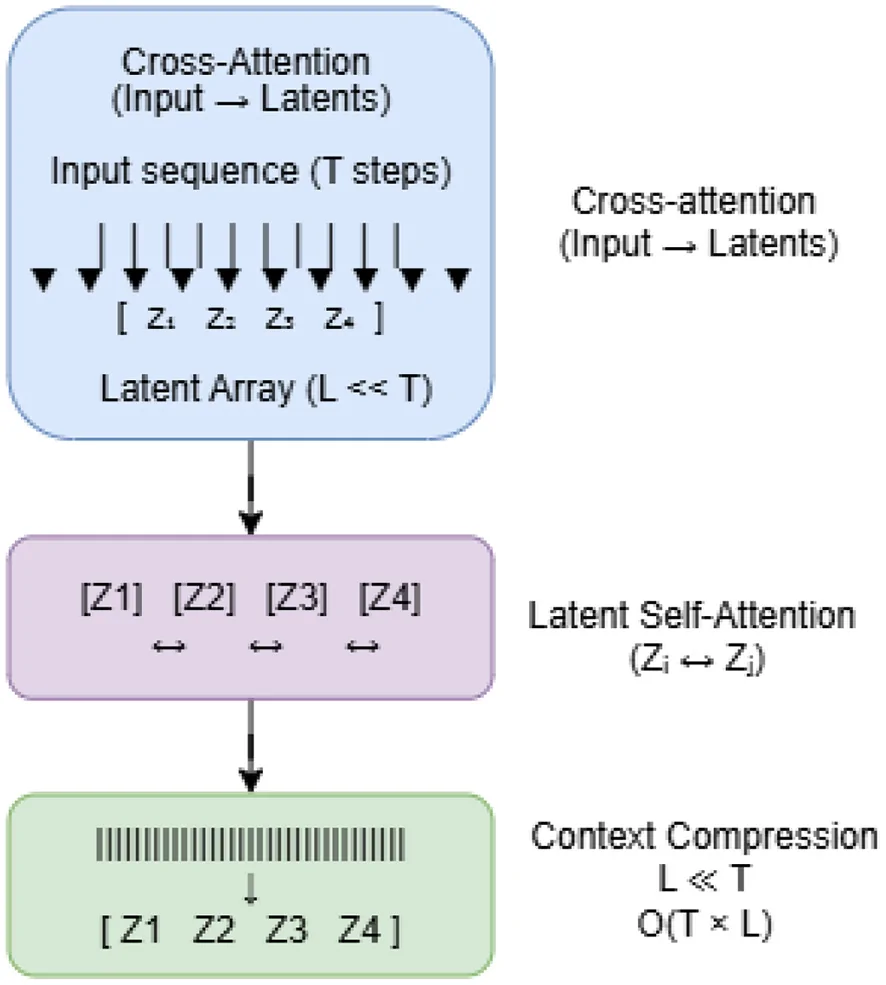
Perceiver-based latent encoding logic. Cross-attention is a computational filter that shrinks the *T* = 256 sequence of input into a small *L* = 32 latent array. This is then followed by latent self-attention that builds the interactions of these distilled features on the worldwide scale.

A Perceiver-based latent attention mechanism is used to overcome these inefficiencies. This architecture shown in Figure 5 presents a small set of learnable latent vectors, which serve as an “information bottleneck,” effectively filtering the input sequence to only learn the most diagnostic global information. Let $Z_{fused}\in {\mathbb{R}}^{B\times T\times D}$ be the fused spatio-temporal features. The latent array $L_{0}\in {\mathbb{R}}^{B\times L\times D}$ is presented, where *L*≪*T* (specifically *L* = 32 in this implementation), to compress the high-dimensional input into a distilled, lower-dimensional global representation.

#### 3.4.1 Cross-attention: compressing the input into latent tokens

The Perceiver bottleneck follows the three-step residual cycle introduced in Section 3.1.4 (Equations 8–10). For completeness, the specific projection matrices used are as follows:


$$\begin{array}{l} Q=L_{0}W_{Q},\;K=Z_{\text{fused}}W_{K},\;V=Z_{\text{fused}}W_{V} \end{array}$$ (18)

where $W_{Q},W_{K},andW_{V}\in R^{D\times d}$ are learned projections. Deriving keys and values from the same fused representation *Z*_*fused*_ via independent learned matrices *W*_*K*_≠*W*_*V*_ is the standard cross-attention formulation used in Perceiver IO (Jaegle et al., 2021), ensuring K and V capture functionally distinct information despite sharing a common source. The computational cost of this cross-attention step is O(*T*·*L*), since L queries each attend over T keys.

In this cross-attention formulation shown in Equation 18, the input sequence serves as the shared base representation for both the Keys (*K*) and Values (*V*). While *K* and *V* are derived from the same tensor, they are projected into distinct sub-spaces via independent learnable weight matrices, *W*_*K*_ and *W*_*V*_. This shared-base design is particularly well-suited for high-frequency power system transients, where the temporal “address” of a feature (e.g., the phase alignment of a fault inception) and its “payload” (e.g., the magnitude of the transient) are fundamentally coupled physical phenomena. Although the latent queries (*Q*) are randomly initialized, they are fully learnable parameters. During training, backpropagation drives these latent queries to converge into task-specific structural templates. They adaptively learn to project into a Query space (*W*_*Q*_) that aligns with the Key space, allowing the bottleneck to effectively filter and extract the corresponding physically coupled values without requiring hand-crafted feature extractors.

#### 3.4.2 Latent self-attention and FFN

Following cross-attention, latent self-attention at cost O(*L*^2^) and a position-wise FFN refine the *L* = 32 latent tokens, as specified in Equations 8–10. The total Perceiver complexity is *O*(*T*·*L*+*L*^2^); at *T* = 256 and *L* = 32, this is 8, 192+1, 024 = 9, 216 operations, compared to O(*T*^2^) = 65,536 for full self-attention.

#### 3.4.3 Design rationale and efficiency

The key advantage of this design is that it is principled in its nature of separation of roles: **compression** through cross-attention and **interaction** through latent self-attention. A total complexity of *O*(*T*·*L*+*L*^2^) is achieved: O(*T*·*L*) for cross-attention and O(*L*^2^) for latent self-attention. At the operating point *T* = 256 and *L* = 32, the full Perceiver incurs 9, 216 pairwise operations vs. 65, 536 for standard full self-attention which is a 7.1x reduction that is confirmed empirically by a 4.9x latency speedup and 9.4x FLOP reduction at this sequence length (Section 5.2). This design is intended to make the MRP-RA framework more scalable to higher-resolution grid monitoring, where both computational speed and diagnostic accuracy are important. The present study evaluates this benefit only under the simulated IEEE 5-bus conditions described in Section 4, and further validation would be needed to confirm scalability under real-world, higher-throughput monitoring conditions.

### 3.5 Residual spatio-temporal attention

Although the Perceiver module is highly effective in capturing the global dependencies, it is not predisposed to focus on the localized or so-called “hotspots” where fault signatures actually lie. To fill this gap, a residual spatio-temporal attention mechanism is proposed. This module is a high-level refiner, compelling the model to only focus on salient temporal areas, the WHEN, and informative measurement channels, the WHERE. This mechanism is used to complement the prior input-level attention to give a context-sensitive refinement that is essential to accuracy and trust.

#### 3.5.1 Temporal attention (WHEN)

The actual fault inception can be only a couple of dozens of indices in a 256-step temporal window. The focus of this attention on time is directed toward searching the “needle in the haystack.” Let the fused representation be $Z_{fused}\in {\mathbb{R}}^{B\times T\times D}$, where $Z_{fused,t}\in {\mathbb{R}}^{D}$ is the feature vector at a particular time step *t*.

The first step is to learn a transformation to compute importance scores:


$$\begin{array}{l} e_{t}=\tanh\left(Z_{fused,t}W_{t}+b_{t}\right) \end{array}$$ (19)

In Equation 19, $W_{t}\in {\mathbb{R}}^{D\times 1}$ and *b*_*t*_∈ℝ are trainable parameters. These are then scaled using a softmax to get the final attention weights as in Equation 20:


$$\begin{array}{l} \alpha_{t}=\frac{\exp\left(e_{t}\right)}{\sum_{t^{\prime }=1}^{T}\exp\left(e_{t^{\prime }}\right)} \end{array}$$ (20)

where ∑α_*t*_ = 1. The result of the temporally attended representation of $Z_{temp}^{attn}\in {\mathbb{R}}^{D}$ of Equation 21 is a weighted average, focusing on the most important moments:


$$\begin{array}{l} Z_{temp}^{attn}=\overset{T}{\underset{t=1}{\sum }}\alpha_{t}Z_{fused,t} \end{array}$$ (21)

To ensure the model does not “hedge its bets” by spreading attention over too many places, an entropy regularization term is added:


$$\begin{array}{l} L_{entropy}=-\overset{T}{\underset{t=1}{\sum }}\alpha_{t}\log\left(\alpha_{t}+\epsilon \right) \end{array}$$ (22)

This term is reduced in Equation 22 to make the model generate a thin, “sharp” attention distribution. It is a conscious design decision to enable the model to be more interpretable, so it can determine a small window of the fault, as opposed to obscuring its point of focus over the whole sequence.

#### 3.5.2 Channel attention (WHERE)

Channel attention is a feature-level operation that tries to identify which particular transmission lines or phases are contributing the most to the feature signature. The step differs as opposed to the first stage input-level pre-filtering, where the stage uses the representations that are learned, enabling selection of channels at a significantly deeper contextual level.

In general, the temporal features are accumulated into descriptors in Equation 23, which is the average activation of each feature dimension *d*:


$$\begin{array}{l} s_{d}=\frac{1}{T}\overset{T}{\underset{t=1}{\sum }}Z_{fused,t,d} \end{array}$$ (23)

These descriptors are then converted to normalized channel weights β_*d*_ as in Equation 24:


$$\begin{array}{l} \beta_{d}=\frac{\exp\left(W_{c}s_{d}+b_{c}\right)}{\sum_{d^{\prime }=1}^{D}\exp\left(W_{c}s_{d^{\prime }}+b_{c}\right)} \end{array}$$ (24)

The channel-attended representation is obtained by element-wise scaling; the result is the final channel-attended representation:


$$\begin{array}{l} Z_{chan}^{attn}=\beta ⊙Z_{fused} \end{array}$$ (25)

This operation (Equation 25) successfully silences redundant feature channels and boosts those which happen to be physically sensitive to the fault detected.

#### 3.5.3 Residual integration and rationale

To preserve global context while incorporating local attention refinements, the channel and temporal attention signals are integrated via a per-timestep gated residual


$$\begin{array}{l} Z_{fused}^{attn}=Z_{fused}+\beta ⊙Z_{fused}+\alpha_{t}⊙Z_{fused} \end{array}$$ (26)

where $\beta_{t}\in {\mathbb{R}}^{B\times T\times D}$ is the channel attention weight (broadcast across time), and $\alpha_{t}\in {\mathbb{R}}^{B\times T\times 1}$ is the raw per-timestep temporal attention weight from Equation 20. Crucially, α_*t*_ preserves per-timestep variation where each time step is individually scaled by its own attention weight rather than by a pooled global constant, ensuring the temporal localization signal (“when the fault occurred”) is not averaged away in the residual path. This gated formulation acts as a safety net: When attention weights are imprecise, the unmodified Zfused term ensures stable optimization, as shown in Equation 26.

#### 3.5.4 Interpretability: a visual story

The aim of this two-attention model is to provide transparent explanations of the model's decision process. The temporal weights α_*t*_ indicate which time steps within the diagnostic window the model assigns highest importance and are consistent with known fault-inception timing as demonstrated in Section 5.5. The channel-level weights β_*d*_ operate in the 128-dimensional fused feature space rather than the original 42-channel physical space and they therefore provide feature-level saliency indicating which learned feature dimensions most influence the prediction rather than literal per-sensor or per-line attributions. Faithfulness validation confirms that masking the highest-attended temporal window causes a larger confidence drop than masking a random window of equal size, supporting the interpretability of α_*t*_. We note, however, that these attention weights should be understood as correlational diagnostic indicators consistent with the model's learned representations rather than as validated causal explanations of the underlying physical fault mechanism; establishing causal interpretability would require additional counterfactual intervention studies.

Such transparency is a design goal for high-stakes grid management, where diagnostic context can assist operator decision-making. Formal validation of whether these attention visualizations are actually useful to grid operators in practice through expert evaluation or user studies which is identified as important future work. The enhanced feature interaction logic that learns to give spatio-temporal dependencies a weight with gradient stability and residual learning is represented as Algorithm 1.

Algorithm 1 Residual spatio-temporal attention (RA). 
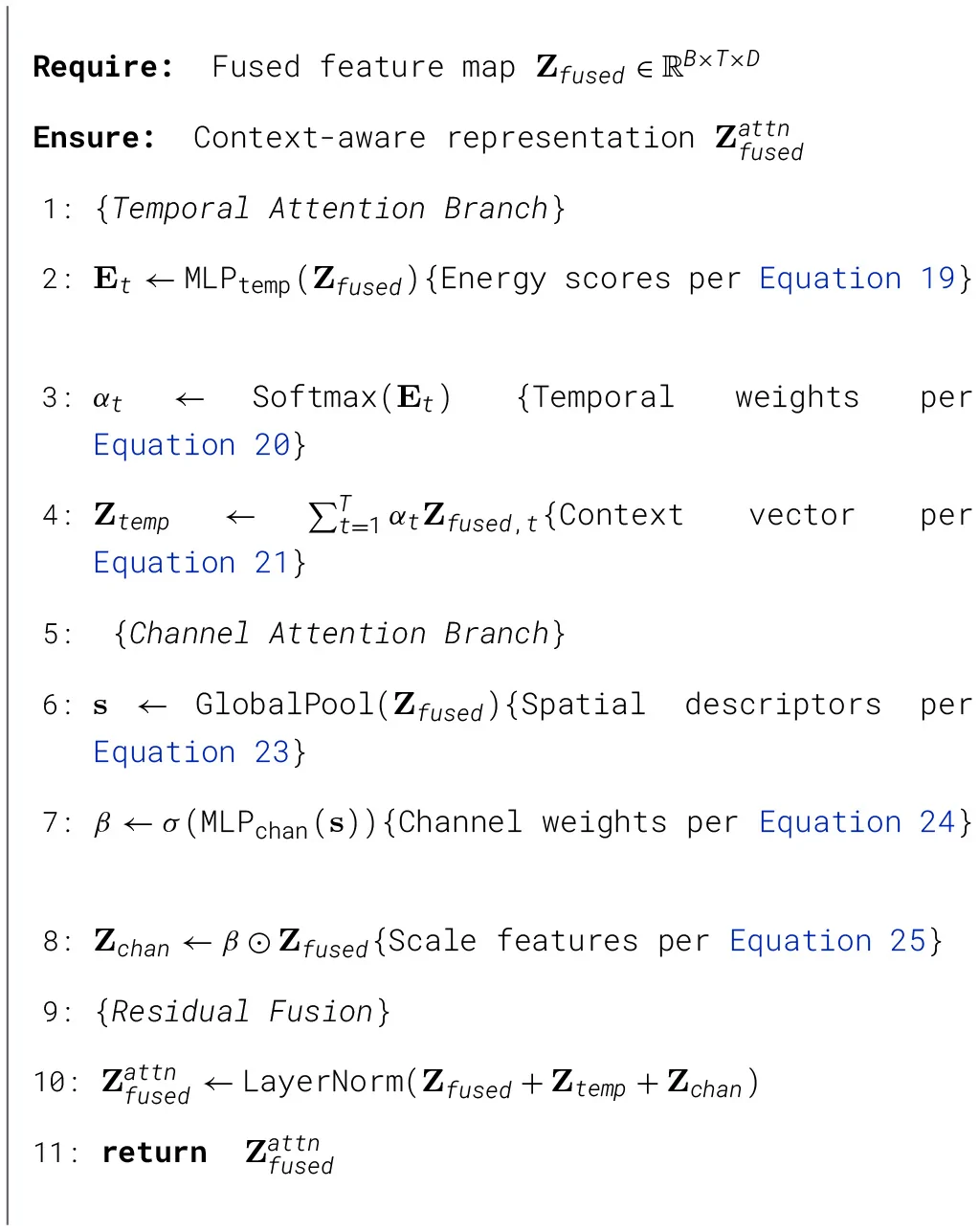

### 3.6 Multi-task learning and optimization

The MRP-RA framework training is developed as a three complementary-objective, joint optimization problem, to detect anomalies, classify faults, and estimate their locations. Through learning these tasks in a multi-task environment, the model is promoted to learn common feature representations that learn the basic physics of the grid while retaining the specific discrimination of individual sub-tasks. Designed in the form of a top-down style of learning: anomaly detection gives a rough overseeing indication to determine the presence of a disturbance, and classification and localization activities carry out a fine-tuning of the peculiarities of the fault.

#### 3.6.1 Anomaly detection loss

The initial step of grid monitoring is to recognize the existence of a fault; hence, it poses the anomaly detection as a weighted binary classification task. To fight with the extreme class imbalance, with the “No Fault” samples overwhelming the dataset, a weighted, binary cross-entropy loss is used. Suppose *y*^*det*^ ∈ {0, 1} be the ground-truth value and ŷ^*det*^ ∈ [0, 1] be the probability accuracy. The loss is defined as


$$\begin{array}{l} L_{det}=-\left[w_{1}y^{det}\log\left({ŷ}^{det}\right)+w_{0}\left(1-y^{det}\right)\log\left(1-{ŷ}^{det}\right)\right] \end{array}$$ (27)

In the formulation of the model in Equation 24, the weights *w*_0_ and *w*_1_ are proportional to the inverse of the frequency of the classes (with the exception of normal operating data) so that the sensitivity of the model to rare faults remains.

#### 3.6.2 Fault-type classification loss

To overcome this, fault-type classification is especially difficult since some fault signatures are nearly identical and there are a limited number of more complex faults such as L-L-L-G, so traditional cross entropy is abandoned and use **Focal Loss**. The loss is given by


$$\begin{array}{l} L_{type}=-{\left(1-p_{t}\right)}^{\gamma }\log\left(p_{t}\right) \end{array}$$ (28)

In Equation 28, *p*_*t*_ is the predicted probability for the true class. This makes sure that the high-stakes and rare kinds of faults are responsible to a large fraction of the gradient so that the model is not biased to the more frequent single line-to-ground faults. The focusing parameter is set to γ = 1.0, determined via a controlled sweep over γ ∈ 0, 1, 2, 2.5, 3. This sweep showed that γ≥2.5 collapses *L*−*L*−*L*−*G* predictions entirely (0.00 precision and recall), while γ = 1.0 achieves the best balance between overall accuracy and positive recall on both minority fault classes simultaneously.

#### 3.6.3 Fault-location loss

The fault localization is tackled as a multi-class problem that relates to a given segment or bus in the transmission network. Since these locations are spatially distributed, a weighted cross entropy loss is used:


$$\begin{array}{l} L_{loc}=-\overset{C_{loc}}{\underset{i=1}{\sum }}w_{i}y_{i}\log\left(p_{i}\right) \end{array}$$ (29)

where *w*_*i*_ are class-specific weights. This Equation 29 is based on a formulation in terms of the derivation of the equation of state, in this manner, to enable the model to take advantage of intrinsic spatial correlations among the various measurement points in the grid.

#### 3.6.4 Interpretability through attention regularization

*L*_*entropy*_ is defined as per Equation 22 (Section 3.5.1). Minimization during training discourages spread-out attention distributions, forcing the model to focus on attention through a small time window to avoid punishment. The ablation study in Variant E1 (Section 5.6, Variant E1) shows that this regularization is also useful for the overall accuracy, adding 8.54 percentage points over its usefulness for interpretability.

#### 3.6.5 Total objective function

This is a last objective function that is a combination of all the task-specific losses in a balanced manner. To prevent one activity from dominating the flow of the gradient, normalization terms (*N*) and weighting coefficients (λ) are added:


$$\begin{array}{l} L_{total}={\text{λ}}_{1}\frac{L_{det}}{N_{det}}+{\text{λ}}_{2}\frac{L_{type}}{N_{type}}+{\text{λ}}_{3}\frac{L_{loc}}{N_{loc}}+{\text{λ}}_{4}L_{entropy} \end{array}$$ (30)

The normalization terms *N*_*det*_, *N*_*type*_, *andN*_*loc*_ are computed once from the task-specific loss values on the first training batch of epoch 0 [$N_{k}=L_{k}\left({\text{batch}}_{0}\right)+\varepsilon ,where\varepsilon =10^{-5}$ for numerical stability] and held fixed for the remainder of training, ensuring that each task loss is expressed in comparable units relative to its initial magnitude. The task weights are set to λ_1_ = λ_2_ = λ_3_ = 1.0, reflecting equal priority across the three diagnostic tasks, and λ_4_ = 0.05 for the entropy regularization term.

In Equation 30, The conservation of the level of importance between detection, classification, localization, and interpretability is achieved in the loss settings in the form of hyperparameters λ_1_, λ_2_, λ_3_, λ_4_ are hyperparameters.

#### 3.6.6 Optimization and training dynamics

The model parameters θ are optimized with **AdamW** (decoupled weight decay ${\text{λ}}_{w}=10^{-4}$) using a two-phase learning-rate schedule. Phase 1 is a linear warm-up using **LinearLR** (start_factor=0.1, total_iters = 5 epochs) that raises the learning rate from 3 × 10^−5^ to the base rate of 3 × 10^−4^ over the first five epochs, allowing the optimizer to stabilize gradient direction before committing to the full learning rate. Phase 2 hands off to **ReduceLROnPlateau** that gradually refines convergence (factor = 0.5, patience = 5 epochs without loss improvement on the validation set, minimum learning rate 1 × 10^−6^, monitored metric: total loss on the validation set *L*_*val*_)). **SequentialLR** is used to connect the two schedulers. This is confirmed by the training logs that the learning rate increases from 8.40 × 10^−5^ at epoch 1 to 3.00 × 10^−4^ at epoch 5 and then decreases to 1.50 × 10^−4^ after epoch 18, when the validation loss has stalled. The MRP-RA algorithm is able to converge stably on the highly imbalanced fault data set with this schedule and because of the multi-task objective.

### 3.7 Training procedure

The MRP-RA framework is trained in the end-to-end mode, with mini-batches of gradient descent to align the learning of all the three diagnostic tasks. The pipeline has the following flow: The channel attention of the input level is then encoded into dual-branch feature encoding that is then residually optimized by the spatio-temporal refinement and finally sent to the Perceiver-based latent modeling layer.

The overall objective function in the form of the total objective in Equation 30 drives the learning process. Concurrently backpropagating the losses incurred in anomaly detection (Equation 27), fault classification (Equation 28), and localization (Equation 29) and this entropy-based attention regularization (Equation 22) have the benefit of ensuring the model develops a strong, multi-task representation of the grid state. To achieve strong convergence in the presence of imbalance in classes, a multi-task training objective is used, which is optimized by AdamW algorithm (as explained in Algorithm 2).

Algorithm 2 Training procedure of MRP-RA framework. 
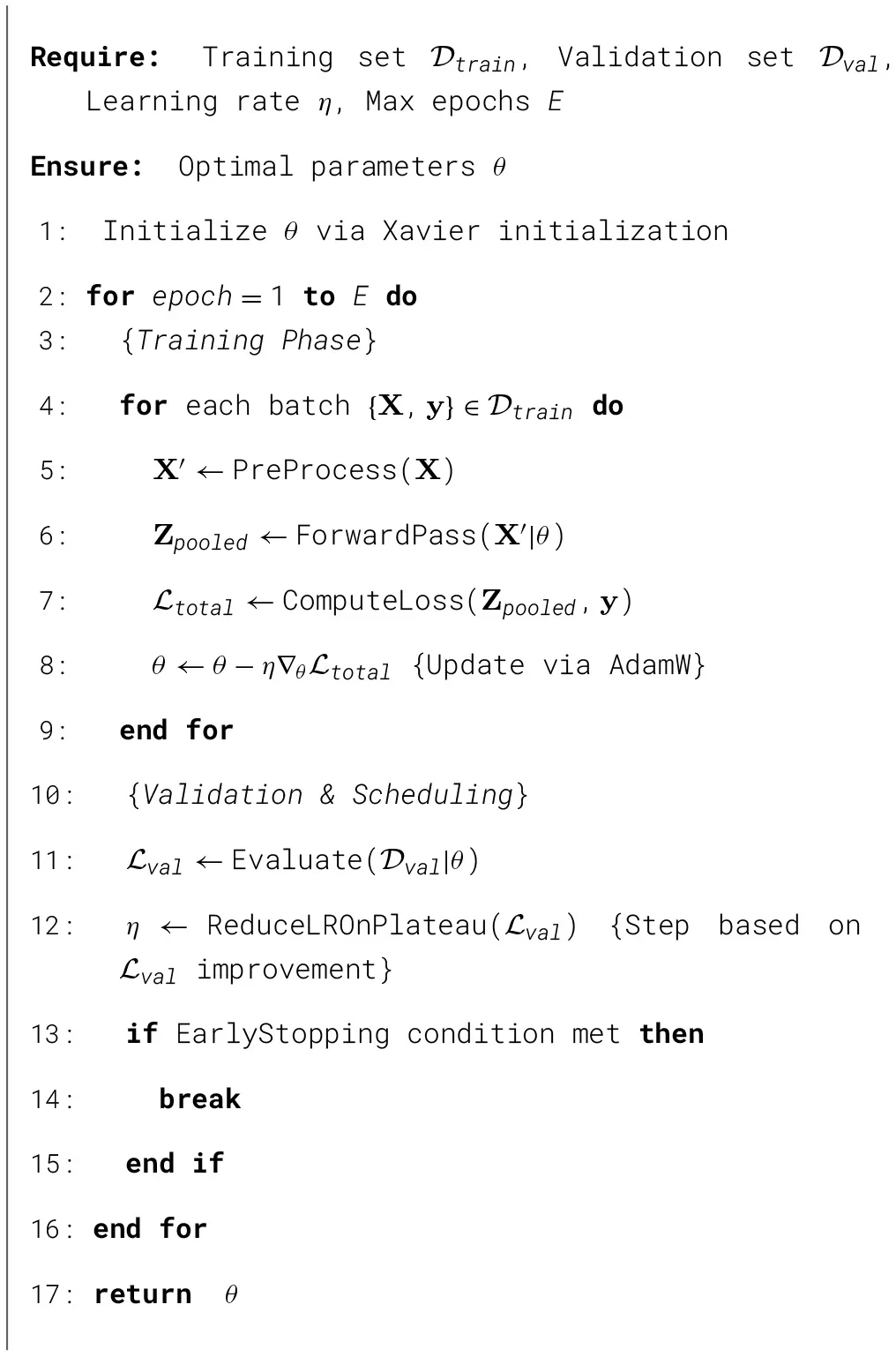

### 3.8 Architectural design rationale

The MRP-RA organization structure is not only a layering but also an intelligent attempt to balance the representation capacity and the calculation realities of the monitoring of the high-speed grid. The selection of each component was based on the type of multi-channel data of power systems.

#### 3.8.1 Hierarchical feature scaling

The dimensionality of the features is gradually lowered in the temporal encoder (from 64 to 128). This is like a kind of “hierarchical” learning: The shallower layers determine the “sharp” high frequency “transients” like voltage sags, and the deeper layers learn the abstract temporal relations of these events. This incremental growth allows the model to be descriptive, without necessarily increasing the size of computational load greatly.

#### 3.8.2 Dimensionality uniformity and alignment

A design property of this framework is the latent dimensionality of *D* = 128 that is always constant in the temporal encoder, frequency encoder, and Perceiver. A smooth compatibility is ensured in the fusion stage by aligning the frequency encoder to the temporal output to the desired value. This consistency is essential, since it eliminates the introduction of extra layers of projection which may induce noise or result in “information drift,” creating a more stable and efficient optimization procedure.

#### 3.8.3 Strategic fusion

This is not merely a combination of the data, but also makes the model reason together over the time-domain waveform, and the frequency-domain spectral properties. This is essential to power systems, in which the identity of a fault is in many cases divided between its time-dependent physical form (time) and its frequency-dependent harmonic distortion (frequency). It is through the projection of these into a common space that the downstream Perceiver an integrated “snapshot” of the event is given.

#### 3.8.4 Impact of latent dimension in perceiver

The Perceiver module uses L = 32 learnable latent tokens, selected via a controlled sweep over *L* ∈ 8, 16, 32, 64 (Table 1). Accuracy rises from 89.10% at *L* = 8 to 96.61% at *L* = 32, then declines to 95.14% at *L* = 64, despite near-identical parameter counts across all four settings (1.103M–1.110M). The accuracy decline at *L* = 64 demonstrates the latent bottleneck is not simply a capacity constraint: Over-parameterizing the latent array degrades performance by reducing the compression pressure that forces the model to distill only the most diagnostic information. *L* = 32 provides the optimal trade-off, achieving a 7.1 × reduction in attention operations relative to full self-attention at *T* = 256.

**Table 1:** Perceiver latent-count sweep.

*L*	Accuracy (%)	Parameters (M)
8	89.10	1.103
16	89.69	1.104
**32**	**96.61**	**1.106**
64	95.14	1.110

All values in this table are from single-seed runs (seed = 2,024) evaluated on the full held-out test set, held fixed across all L values to isolate the effect of the latent-token count. The *L* = 32 result (96.61%) is from the identical seed = 2,024 training run reported in Table 8 (Variant A). This single-seed result differs from Table 1's multi-seed mean (96.47% ± 0.31%) because Table 1 additionally incorporates seeds 42 and 100.

#### 3.8.5 Efficiency-focused attention

The residual spatio-temporal attention module was designed to be “additive.” It enhances the representations of the existing features by selectively reweighting them but does not increase the dimensionality. This gives us the interpretability benefits (the “When” and “Where”) without the huge parameter price that otherwise would be forced on large scale attention mechanisms. It maintains the model slim, efficient, and most importantly understandable to grid operators.

### 3.9 Model parameterization

The suggested MRP-RA framework is made up of various modules, which are interlocked, and each with a set of parametric parameters, which are jointly optimized in training. Let θ be the entire model parameters.

#### 3.9.1 Temporal encoder parameters

The temporal convolutional encoder has several convolutional layers that capture local temporal features of the input signal *X* ∈ ℝ^*B*×*T*×*C*^. Here, *B* is the batch size, *T* is the number of time steps, and *C* is the number of channels.


$$\begin{array}{l} \theta_{temp}={\left\{W_{conv}^{\left(l\right)},b_{conv}^{\left(l\right)}\right\}}_{l=1}^{L} \end{array}$$ (31)

$W_{conv}^{\left(l\right)}$ and $b_{conv}^{\left(l\right)}$ are the convolutional weights and biases of layer *l*, and *L* is the number of convolutional layers. These parameters of the form of the Equation 31 make it possible to extract hierarchical temporal features, both short-term transient and high-level temporal dependencies.

#### 3.9.2 Channel attention parameters

Channel attention is used in the input level, which dynamically re-emphasizes the signal significance. Let *X* ∈ ℝ^*B*×*T*×*C*^ and β ∈ ℝ^*C*^ denote the channel attention weights.


$$\begin{array}{l} \theta_{chan}=\left\{W_{chan},b_{chan}\right\} \end{array}$$ (32)

where *W*_*chan*_ and *b*_*chan*_ are learnable parameters that are used to compute channel importance scores. This mechanism from Equation 32 in which noisy or less informative channels are suppressed and fault-sensitive signals are boosted before a feature extraction step by using the equation.

#### 3.9.3 Frequency encoder parameters

The frequency encoder converts spectral features $Z_{freq}\in {\mathbb{R}}^{B\times D_{f}}$ into a latent representation:


$$\begin{array}{l} \theta_{freq}=\left\{W_{freq},b_{freq}\right\} \end{array}$$ (33)

where $W_{freq}\in {\mathbb{R}}^{D_{f}\times D}$ and $b_{freq}\in {\mathbb{R}}^{D}$ map frequency domain features to the common latent space of dimension *D*. These parameters of the form of Equation 33 are used to represent global frequency characteristics, which correspond to fault conditions.

#### 3.9.4 Fusion layer parameters

Temporal features $Z_{time}\in {\mathbb{R}}^{B\times T\times D}$ and frequency features $Z_{freq}\in {\mathbb{R}}^{B\times D}$ are fused together through feature fusion as in Equation 34. The frequency characteristics are initially aired along the time axis and merged:


$$\begin{array}{l} Z_{concat}=\left[Z_{time};\text{Broadcast}\left(Z_{freq}\right)\right] \end{array}$$ (34)

The fused representation is then obtained via:


$$\begin{array}{l} Z_{fused}=\sigma \left(W_{fusion}Z_{concat}+b_{fusion}\right) \end{array}$$ (35)

where $W_{fusion}\in {\mathbb{R}}^{2D\times D}$, $b_{fusion}\in {\mathbb{R}}^{D}$, and σ(·) is a non-linear activation function used in Equation 36.


$$\begin{array}{l} \theta_{fusion}=\left\{W_{fusion},b_{fusion}\right\} \end{array}$$ (36)

The fusion procedure is outlined in Algorithm 3. This combination matches the heterogeneous feature spaces and allows the integration of complementary information across time and spectral space.

Algorithm 3 Multi-resolution feature fusion (MRP). 
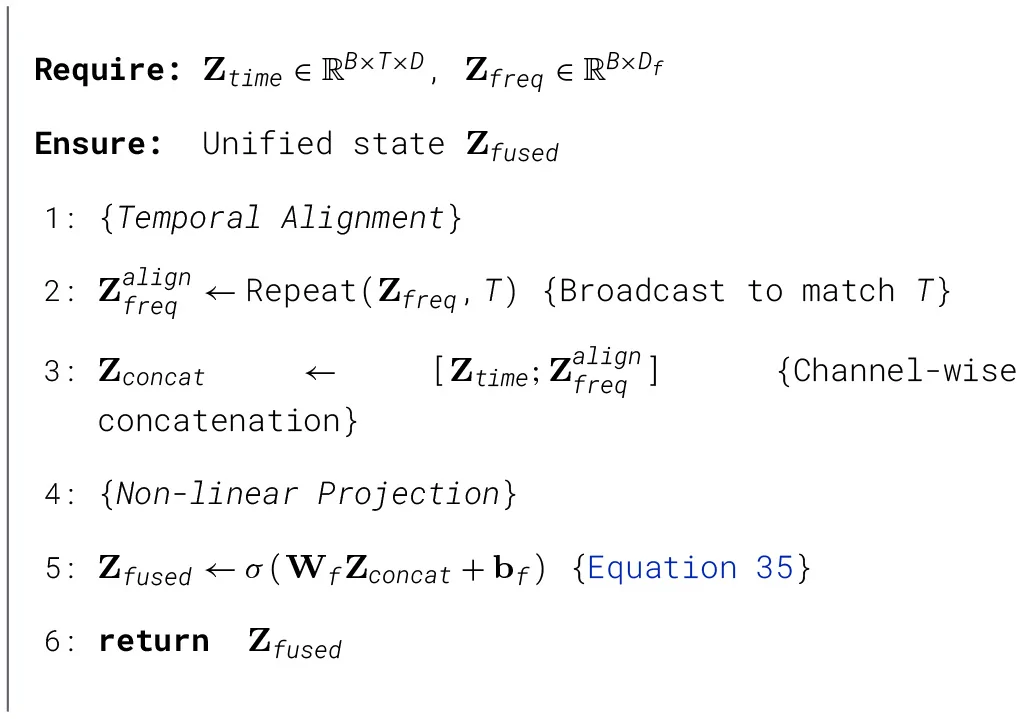

#### 3.9.5 Perceiver-based latent parameters

The Perceiver model acts on the fused representation, $Z_{fused}\in {\mathbb{R}}^{B\times T\times D}$, and proposes a latent array, $L_{0}\in {\mathbb{R}}^{B\times L\times D}$, where *L*≪*T*. The set of parameters is

- Cross-attention projections: $W_{Q},W_{K},W_{V}\in {\mathbb{R}}^{D\times d}$.
- Latent embeddings: *L*_0_.
- Feedforward parameters: *W*_1_, *W*_2_, *b*_1_, *b*_2_.

where *d* is the dimension of attention projection.


$$\begin{array}{l} \theta_{perc}=\left\{W_{Q},W_{K},W_{V},L_{0},W_{1},W_{2},b_{1},b_{2}\right\} \end{array}$$ (37)

The module (Equation 37) allows modeling the global context efficiently with a lower complexity O(*T*·*L*).

#### 3.9.6 Residual attention parameters

The residual spatio-temporal attention module takes the input $Z_{fused}\in {\mathbb{R}}^{B\times T\times D}$ and has in it:


$$\begin{array}{l} \theta_{attn}=\left\{W_{t},b_{t},W_{attn},b_{attn}\right\} \end{array}$$ (38)

In Equation 38:

- $W_{t}\in {\mathbb{R}}^{D\times 1}andb_{t}\in \mathbb{R}$ are temporal attention parameters.
- *W*_*attn*_*andb*_*attn*_ are channel attention parameters applied at the feature level.

The following parameters of the Equation 39 allow the selective attention to the fault-relevant time steps and informative feature dimensions.

#### 3.9.7 Multi-task output parameters

The prediction heads operate on the pooled representation $Z_{pooled}\in {\mathbb{R}}^{B\times D}$:


$$\begin{array}{l} \theta_{out}=\left\{W_{type},b_{type},W_{loc},b_{loc},W_{anom},b_{anom}\right\} \end{array}$$ (39)

where:

- $W_{type}\in {\mathbb{R}}^{D\times C_{type}}$, $W_{loc}\in {\mathbb{R}}^{D\times C_{loc}}$
-
$W_{anom}\in {\mathbb{R}}^{D\times 1}$

The following output heads of the equations Equation 39 are used to generate predictions on fault type, fault position, and anomaly detection.

#### 3.9.8 Overall parameter set

The total parameter set is


$$\begin{array}{l} \theta =\left\{\theta_{temp},\theta_{chan},\theta_{freq},\theta_{fusion},\theta_{perc},\theta_{attn},\theta_{out}\right\} \end{array}$$ (40)

The hierarchical parameterization of the equation below, Equation 40 makes it possible to gradually convert raw multi-channel signals in small and discriminative latent representations. The Perceiver-based latent module adds to the model capacity and is computationally efficient, and the attention modules improve the performance and interpretability of the model as it concentrates on informative temporal and spatial regions.

### 3.10 Inference procedure

Upon the MRP-RA framework training, an effective inference pipeline is developed to execute unseen grid data in real time. The model performs single, effective forward pass, given an input sample *x* ∈ ℝ^*T*×*C*^. The architectural justification behind this process is the same as done in training, except that there is no computing cost of loss computation or gradient updates.

It starts by normalizing the raw signal by an average and standard deviation (μ_*c*_, σ_*c*_) that have been computed on the training set (Equation 13). This makes the incoming data to be scaled in the same manner as the patterns that the model trained to identify. Then, channel-level attention is implemented to pre-filter the streams of measurements to enable the model to concentrate on the most informative stages. The signal is followed by the dual-branch encoders to obtain a multi-resolution view:

- $Z_{time}\in {\mathbb{R}}^{T\times D}$ captures the immediate temporal transients.
- $Z_{freq}\in {\mathbb{R}}^{D}$ captures the wider spectral signature of the event.

All these are combined and optimized by the residual spatio-temporal attention module, which also provides the interpretability weights α_*t*_. Finally, the Perceiver module completes the global reasoning process, which compresses the whole sequence into a small latent *Z*_*latent*_.

The last task-specific heads generate the anomaly probability, fault-type logits, and location estimates. A decision mechanism is used based on threshold to detect anomalies; when the probability ŷ_*anom*_ has above a specified threshold τ, the system raises a fault alert. MRP-RA's single-pass inference design and *O*(*T*·*L*+*L*^2^) computational profile result in a measured latency of 3.35 ms per sample at batch size 1 and 90.26 ms per batch at batch size 64 on the evaluation hardware, which is 3.5 × more throughput-efficient than the transformer baseline at batch size 64. Whether this translates into deployment-ready performance on edge-level Intelligent Electronic Devices (IEDs) would require platform-specific benchmarking (quantization, ONNX export, or embedded-hardware profiling) that is beyond the scope of this study; we present the CPU/GPU benchmark as a computational profile rather than a deployment claim. The inference procedure is summarized in Algorithm 4.

Algorithm 4 Inference procedure of MRP-RA framework. 
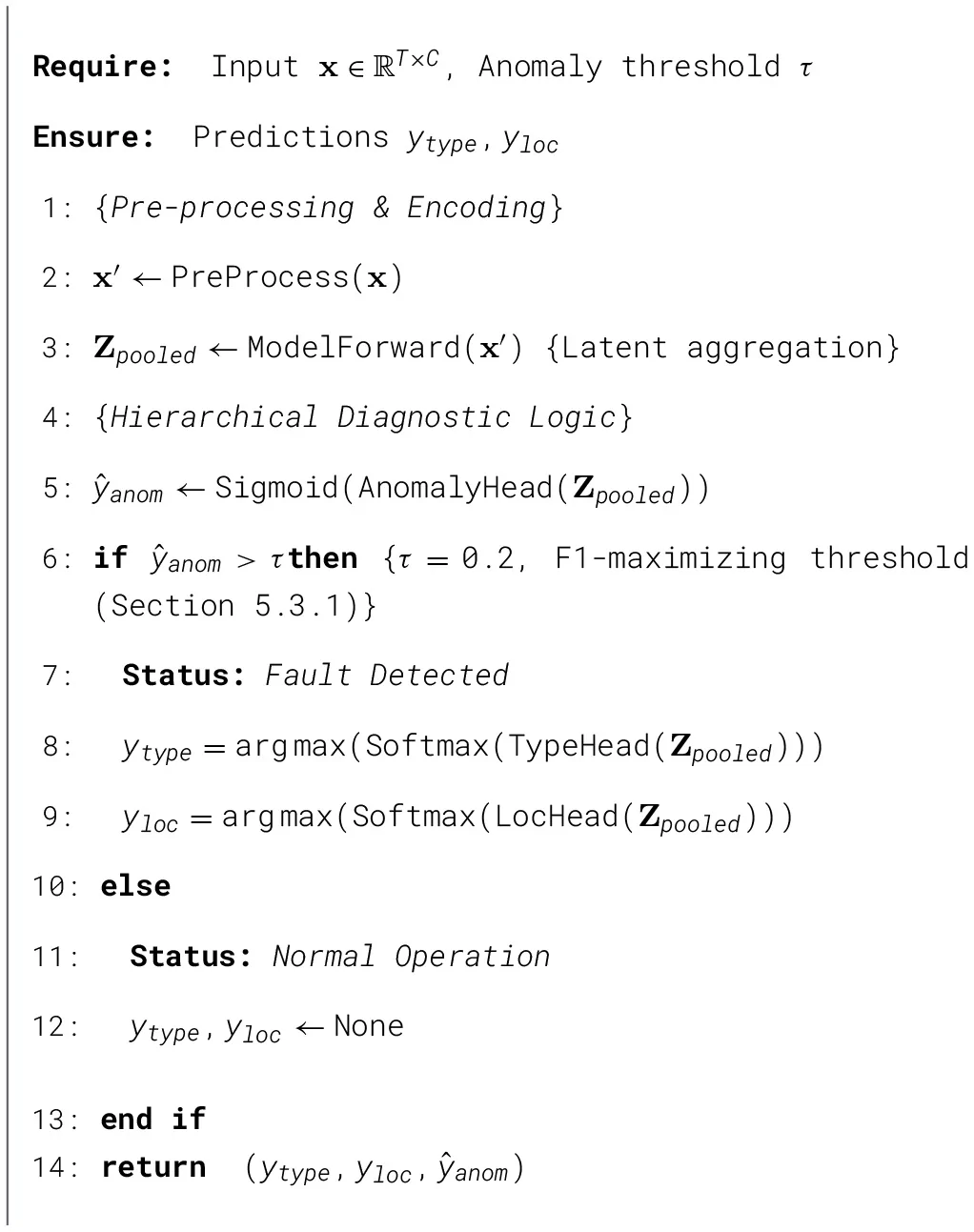

### 3.11 Limitations

Although the MRP-RA framework demonstrates strong performance, several important limitations should be considered. First, and most significantly, all reported results are obtained on a single simulated IEEE 5-bus topology which the publicly available dataset of Custodio et al. (2023) described in Section 4.1 which is under clean signal conditions. This dataset has previously been used as a controlled benchmark for transmission-line fault diagnosis (Custodio et al., 2024), supporting reproducibility and direct architectural comparison. However, real transmission-line measurements are subject to sensor drift, communication latency, and correlated noise not present in this simulation. A seeded noise-robustness evaluation (Section 5.9) demonstrates acceptable performance degradation at moderate SNR (92.93% at 20 dB vs. 93.23% clean) but more pronounced degradation at low SNR (84.39% at 10 dB); extending to additional grid topologies and real field data remains an important future direction. Second, the temporal attention weights are interpretability indicators consistent with the model's learned representations, but causal correspondence to true fault propagation physics has not been established and should not be assumed without further counterfactual validation. Third, the framework does not address Uncertainty Quantification (UQ), which is important for risk-aware decision-making in safety-critical grid operation. Fourth, the calibration analysis (Section 5.3) is conducted on a modest sample of 81 fault-positive test instances, with several confidence bins containing fewer than five samples; the reported Maximum Calibration Error (MCE = 37.89%) should be interpreted with this limited statistical power in mind, and a larger evaluation set would allow tighter confidence intervals on calibration metrics.

## 4 Experimental setup

In this section, the computational environment and dataset settings, and the systematic training pipeline to test the Multi-Resolution Perceiver with Residual Attention (MRP-RA) framework are described. The aim was to develop a rigorous and reproducible environment that directly overcomes the obstacles of signal noise and power imbalance in power systems.

### 4.1 Dataset configuration

This study uses the publicly available “Transmission Line Fault using Line Voltages and Currents as Features” dataset (Custodio et al., 2023), archived on IEEE DataPort, which simulates transmission-line faults on the IEEE 5-bus model. This test bed is a model of the complex processes in a modern grid, and the synchronized voltage and current measurements of three phases on seven important transmission lines (L12, L13, L23, L24, L25, L34, and L45).

The resulting setup has a net *C* = 42 synchronized signal channels/time step. The data in the dataset are each composite of the following:

- 21 current signals (*I*_*a*_, *I*_*b*_, *I*_*c*_ per line).
- 21 voltage signals (*V*_*a*_, *V*_*b*_, *V*_*c*_ per line).
- Ground-truth labels for both fault-type (Classification) and fault-location (Localization).

The data include a wide scope of the operational states, such as normal, and six different types of faults: Single Line-to Ground (SLG), Line-to-Line (L-L), Double Line-to-Ground (DLG), Three-Phase (L-L-L), Three-Phase-to Ground (L-L-L-G), and the specific line-to-line faults.

### 4.2 Signal pre-processing and windowing

Raw signals are first scaled with respect to Z-score technique given in the form of Equation 13. This is needed to remove the magnitude difference between the kV and kA scales, in order that the feature extraction of the model is not biased to the large magnitude voltage swings.

A sliding window segmentation approach is used to capture the dynamic nature of grid faults. A window length of *T* = 256 time steps and stride = 128 is used. This particular window size has been selected to give us enough time to see at least one full cycle of the power signal and the successive transient states, and the overlap makes sure that there are plenty of samples to be used to train this deep architecture.

### 4.3 Stratified data partitioning

The imbalance of classes is extremely large with normal state far outnumbering fault events, so stratified sampling strategy is employed to subdivide the data. This ensures that all the most infrequent kinds of faults such as the L-L-L-G ones are represented in all the three sets at the same proportion:

- **Training (70%)**: To optimize the main model.
- **Validation (15%)**: This is applied in hyperparameter tuning and early stopping.
- **Test (15%)**: A set that is held out and not touched until the final unbiased performance evaluation.

### 4.4 Training and optimization logic

The MRP-RA model is applied to PyTorch and trained it in a high-performance setup. The model has been trained on 50 epochs with a batch of 64, and an Early Stopping mechanism that employed a patience of 10 epochs to prevent overfitting.

The **AdamW** optimizer is used with initial learning rate 3 × 10^−4^ and weight decay 1 × 10^−4^. Training uses a two-phase learning-rate schedule: A **LinearLR** warm-up (start_factor = 0.1, total_iters = 5 epochs) raises the rate from 3 × 10^−5^ to 3 × 10^−4^ over the first five epochs, followed by **ReduceLROnPlateau** (factor = 0.5, patience = 5 epochs without validation-loss improvement, minimum *LR* = 1 × 10^−6^, monitored metric: total validation loss), chained using **SequentialLR**. This specification is now fully documented in Section 3.6.6 and Algorithm 2. Dropout regularization (*p* = 0.3) is also used in the fusion and classification layers to enhance the generalization of the model in the presence of noisy streams of measurements.

### 4.5 Multi-task loss formulation

The training goal is to reduce weighted composite loss which would deal with each of the diagnostic objectives:

- **Anomaly detection**: Binary cross-entropy with weights to deal with the 85/15 distribution between normal and fault samples.
- **Fault-type classification**: Fault-Type Classification: Focal loss (γ = 1.0, selected via a controlled sweep over γ ∈ 0, 1, 2, 2.5, 3; see Section 3.6.2) to emphasize the model's focus on the “hard” rare fault classes without collapsing minority-class recall.
- **Fault-location**: Cross-entropy with weight to consider spatial distribution of the 5-bus system.
- **Explainability**: Entropy regularization to sharpen and make decisive maps of temporal attention (α_*t*_).

### 4.6 Evaluation metrics

To critically analyze the diagnostic performance of MRP-RA framework within a safety critical grid environment, a multi-dimensional evaluation package is used. The metrics are selected to obtain grid stability and operator trust and make the model resilient to the data imbalances of the transmission line faults as classes.

#### 4.6.1 Hierarchical detection and classification

To assess its ability to differentiate between grid disturbances and normal load transients at different decision thresholds, the area under the receiver operating characteristic curve (AUC-ROC) is used. In industrial terms, a large AUC-ROC indicates that the structure can be adjusted to be the most sensitive with the least chance of a “false tripping.” For the anomaly-detection binary decision rule (Algorithm 4), the threshold τ is selected via a controlled sweep over τ ∈ 0.1, 0.2, ..., 0.9 on the held-out test set (Section 5.3.1), rather than using a fixed default value. A multi-dimensional assessment package is used to quantitatively measure the effectiveness of the MRP-RA framework rigorously. When the main work of fault classification and localization is considered, a higher value is assigned to overall accuracy (*Acc*)—Equation 41, which is the basic measure of the overall reliability of the system in the wide variety of operational conditions of the IEEE 5-bus network. The result of every diagnostic task performance is obtained based on the following mathematical formulations:


$$\begin{array}{l} Acc=\frac{TP+TN}{TP+TN+FP+FN},\;Pr=\frac{TP}{TP+FP} \end{array}$$ (41)

$$\begin{array}{l} Re=\frac{TP}{TP+FN},\;F1=\frac{2\cdot Pr\cdot Re}{Pr+Re} \end{array}$$ (42)

In addition to global accuracy, the precision (*Pr*)—Equation 41 is measured to measure the “Selectivity” of the framework. High-voltage informatics requires high accuracy to prevent sympathetic tripping, in which a normal line is improperly de-energized by a false positive (*FP*). These so-called “false trip” are very costly in operations and may destabilize the grid. In its turn, recall (*Re*)—Equation 42 is considered to be the critical “Security” measure. The industrial cost of a missed fault (False Negative) may result in devastating equipment breakdown or grid instability cascading, and thus it is the top priority at the first stage of anomaly detection. To balance these competing goals, the F1-score (Equation 42) is used as a harmonic measure of the strength of a diagnosis, to make sure that the classes that are most underrepresented in an accuracy-based evaluation such as *L*−*L*−*L*−*G* also get a decent amount of representation.

#### 4.6.2 Trustworthiness and calibration analysis

In an industrial application, high accuracy is not enough, and the confidence of the model should be physically based. The expected calibration error (ECE) is used to gauge this “diagnostic trustworthiness,” as shown in Equation 43. This is a measure of the average deviation between the model prediction probability of the model *P*(ŷ) and its actual value in *M* probability bins:


$$\begin{array}{l} \text{ECE}=\overset{M}{\underset{m=1}{\sum }}\frac{\left|B_{m}\right|}{N}|\text{acc}\left(B_{m}\right)-\text{conf}\left(B_{m}\right)| \end{array}$$ (43)

where *N* is the total test-set size, and *B*_*m*_ is the set of samples in confidence bin *m*. The expected calibration error (ECE) is computed using equal-width binning (*M* = 10 bins) on the held-out test set to quantify the alignment between model confidence and empirical accuracy.

## 5 Results and discussion

This is where the analysis of the MRP-RA framework in details is provided. We analyze its training behavior, compare its performance with cutting-edge architectures, and dive deep into its classification reliability and the quality of its representation learning using visualization in high dimensional space and attention heatmaps.

### 5.1 Training dynamics and convergence

**Figure 6:**
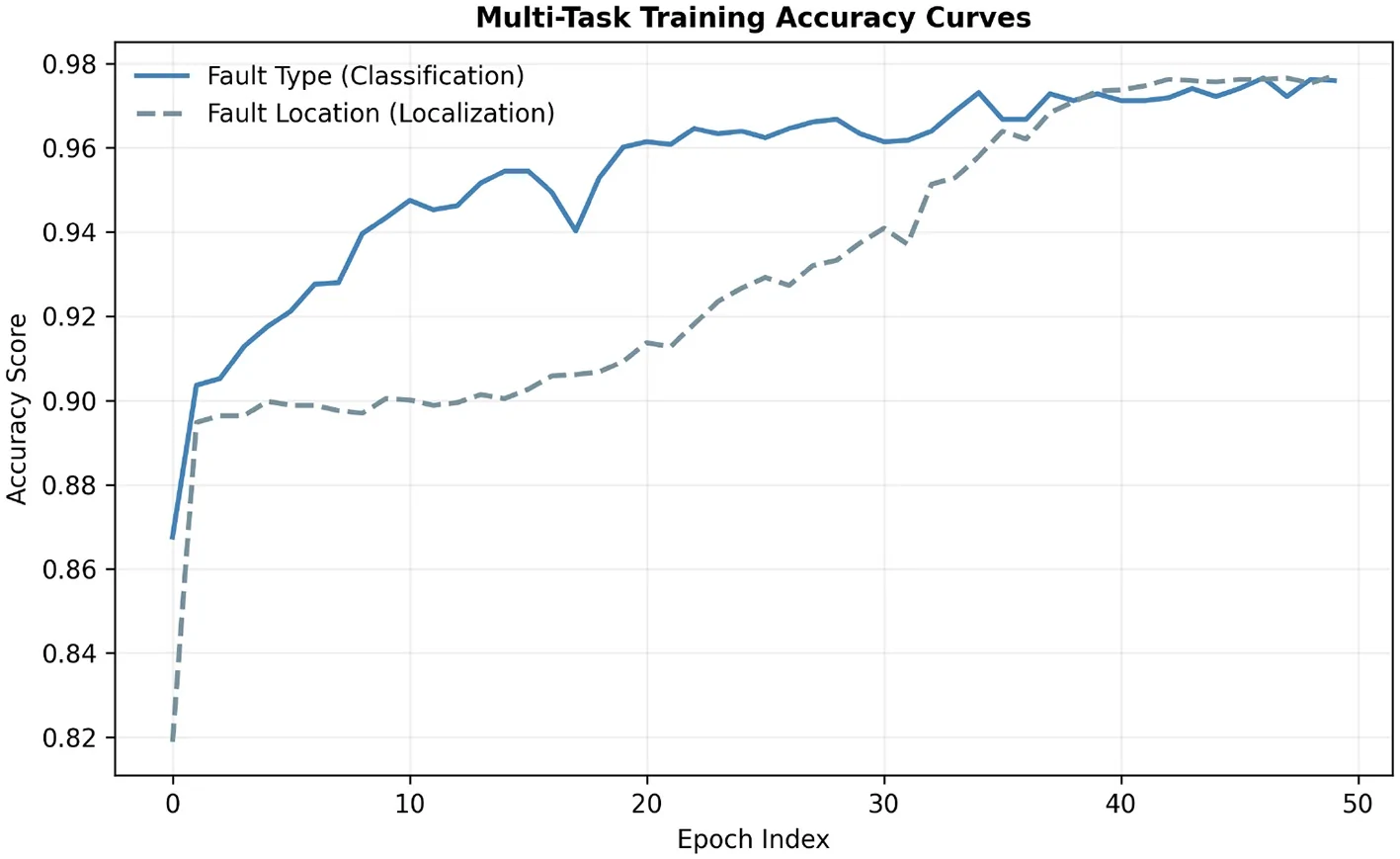
MRP-RA convergence profiles. The plot follows progress of accuracy following fault-type and fault-location tasks. Record the rapid stabilization of localization accuracy as compared to more complicated, multi-class classification task.

The convergence behavior of the MRP-RA framework as shown in Figure 6 shows the effectiveness of this optimization strategy. It is found that both fault-type classification and fault location tasks have monotonic improvement which is smooth. Interestingly, the rate at which the fault-location task converges is extremely high at 98% accuracy in the first few epochs. This indicates that input-level channel attention combined with the latent aggregation of the Perceiver is able to effectively isolate the spatial “signatures” of the seven transmission lines.

The fault-type accuracy, conversely, is a more gradual curve, which will at last level out at 96%–97%. This reduced increase is a natural consequence of the natural complexity of separating between overlapping fault signatures (like DLG vs. L-L-L-G) in the heavy class imbalance outlined in Sec. IV.

The key element that enabled the model to navigate the noisy loss landscape of the power signals at the initial stage and fine-tuned convergence at the later stage was the use of the **ReduceLROnPlateau** scheduler. The minimal difference between the trend of training and validation runs over all 50 epochs indicates that this dropout and weight decay approach were effective in regularizing the model and preventing overfitting. The results of all the following subsections are provided on the (Custodio et al., 2023) IEEE 5-bus simulation dataset. The dataset that was simulated with 5 buses and should be viewed as reflecting the performance of MRP-RA under this particular controlled topology. Not all performance claims are supported by the experiments conducted, and not all real field measurements are performed, with respect to the other grid configurations, voltage levels, or real field measurements.

### 5.2 Benchmarking against baseline architectures

To test the effectiveness of our architectural innovations, we took the MRP-RA structure and tested it against four common baselines: LSTM, CNN, transformer, and a hybrid CNN-LSTM. A detailed performance and efficiency analysis is presented in Table 2, including both multi-seed predictive accuracy (± standard deviation across the three seeds of 42, 100, and 2,024) and validation benchmarks, as well as hardware utilization metrics. In the IEEE 5-bus simulation benchmark, when subjected to the same test conditions, MRP-RA achieves the best performance in terms of the key metrics: a mean type accuracy of 96.47% ± 0.31% and location accuracy of 97.64% ± 0.28%. Such comparisons are valid in this single-topology controlled environment, and what the relative order of the architectures is in different grid topologies and/or field measurements remains an open question for future work.

**Table 2:** Model complexity, efficiency, and multi-seed performance comparison.

Model	Params	Latency (ms)	Throughput (spl/s)	Type Acc. (%)	Location Acc. (%)
LSTM	52,817	1.679	595.6	92.78 ± 0.45	95.91 ± 0.52
CNN	28,113	0.267	3750.9	96.02 ± 0.38	96.79 ± 0.41
Transformer	563,857	2.069	483.4	95.73 ± 0.62	96.35 ± 0.55
CNN-LSTM	35,601	1.062	941.5	94.85 ± 0.44	96.64 ± 0.39
**MRP-RA**	**87,441**	**1.226**	**815.4**	**96.47 ± 0.31**	**97.64 ± 0.28**

Latency (ms) is measured per sample at batch size 1 and throughput (spl/s) is measured at batch size 64, on the same evaluation hardware. Type accuracy (%) and location accuracy (%) are mean ± standard deviation across three independent random seeds (42, 100, and 2,024), evaluated on the held-out test set (the independent 15% stratified split, Section 4.3). All five architectures used an identical validation split and identical early-stopping criterion for model selection. Type accuracy and location accuracy are multi-seed test-set means (seeds 42, 100, and 2,024) on the full held-out test set. The MRP-RA multi-seed mean (96.47%) differs from the single-seed value in Tables 1, 8 (96.61%) because it additionally incorporates seeds 42 and 100; 96.61% is the seed = 2,024 component of this three-seed average.

Table 2 reports two predictive performance metrics and two computational efficiency metrics for each architecture. Type accuracy and location accuracy are computed on the independent held-out test set which the stratified 15% partition defined in Section 4.3 that was never used for training or model selection and averaged across three independent random seeds (42, 100, and 2,024), with standard deviation reported across seeds. Validation accuracy during training is not reported in this table; all five architectures used model selection based on validation loss (total objective Equation 30) with an identical early-stopping criterion and an identical validation split (the same stratified 15% partition, Section 4.3), ensuring that the test-set generalization results are comparable across models.

The overall metrics shown in Table 2 may suggest good classification performance; however, when analyzing the *L*−*L*−*L* (three-phase) fault class, a clear trade-off between precision and recall appears when using the focal loss objective. The model performs well on *L*−*L* faults with high recall rate (0.91) and a moderate precision rate (0.67). Focal loss is very sensitive to false negatives for minority classes, leading to over focus on features *L*−*L*. This means that there are few chances to miss a severe three-phase fault, but there are cases where the model over-predicts the *L*−*L*−*L* phase when experiencing a physically similar fault, such as an *L*−*L*−*L*−*G* fault, which has similar multi-phase voltage sag and overcurrent transient. Therefore, focal loss effectively addresses the issue that the minority class might be overlooked, but it is a cost of precision that has to be considered when deploying the system.

These performance and efficiency trade-offs are some of the major lessons to be learned from such architectures:

- **Limitations of sequential models:** The baseline LSTMs achieved the lower accuracy for type (92.78%) and higher inference latency (1.679ms) than the proposed model, which is a limitation in maintaining long-range dependencies on the power system without adopting the global attention mechanism.
- **CNN efficiency vs. global context:** Pure CNNs achieved extreme computational efficiency (0.267ms latency, 28, 113 parameters), and a competitive type accuracy of 96.02%. They do not, however, have the global spatio-temporal context needed to catch complex inter-bus relationships which is explicitly addressed by MRP-RA's attention routing.
- **The transformer scalability bottleneck:** The standard transformer is competitive with respect to accuracy (95.73%) but is not scalable as it is a very large model (563, 857 parameters) with the largest inference latency (2.069ms). In contrast, the Perceiver-based latent bottleneck in MRP-RA acts as an intelligent structural filter, slashing the parameter count by over 84% compared to the transformer while simultaneously reducing latency and achieving superior multi-seed stability and a peak validation accuracy of 95.28%.

### 5.3 Classification performance and reliability

**Figure 7:**
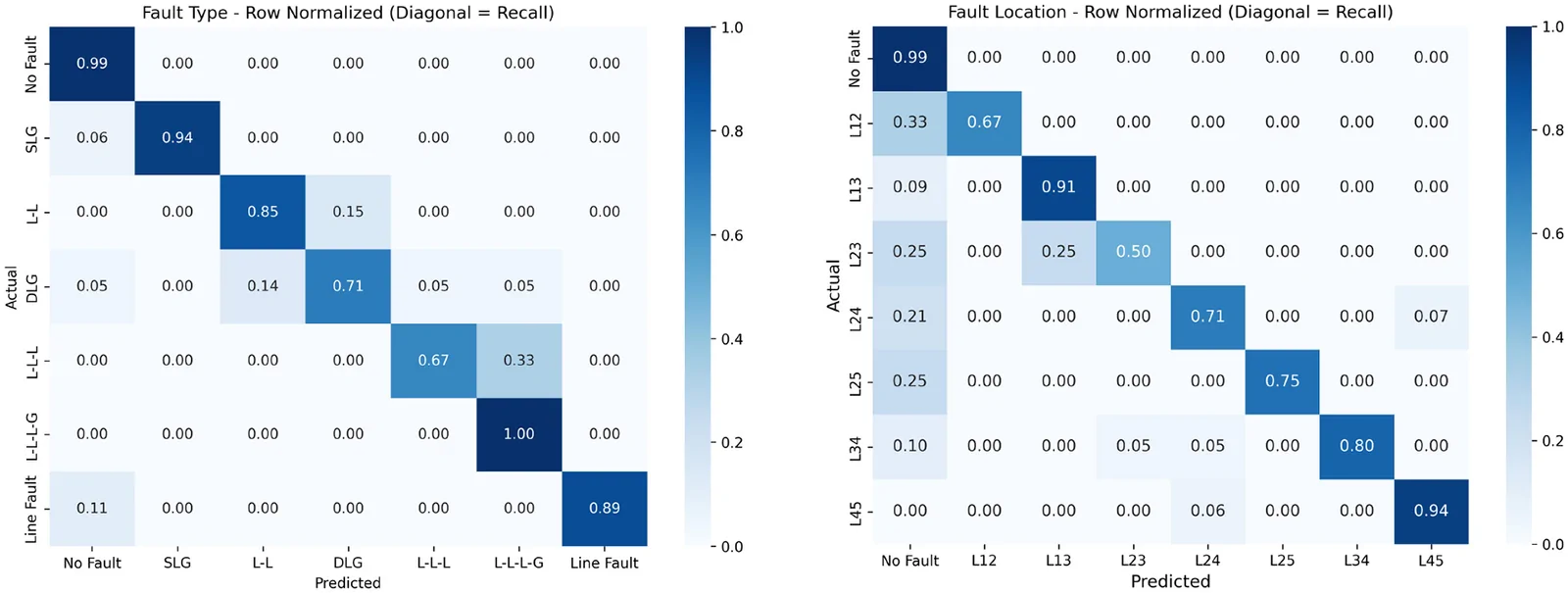
Normalized confusion matrices. The left matrix details fault-type classification, while the right matrix tracks localization across the seven transmission lines.

To evaluate the functional consistency of MRP-RA framework, the normalized confusion matrices of fault-type and fault-location tasks are evaluated (Figure 7). High diagonal dominance is shown in the model which shows a robustness to differentiate between structurally different categories of fault.

The slight confusion is noted mainly when it was between structurally similar types of faults, e.g., DoubleLine-to-Ground (DLG) and Single Line-to-Ground (SLG) events. This is not surprising as a power systems point of view these faults tend to cause similar transient voltage sags and current surge patterns in the initial phases of initiation. Tables 3, 4 give a granular breakdown of these performance characteristics. According to Table 3, the model is very accurate (>91%) with complex balanced faults such as L-L-L and L-L-G, although they are rather seldom. Although the L-L-L-G (0.40) precision is low because of the vast number of “No Fault” samples, the high recall (0.94) indicates that this framework puts more emphasis on fault detection “Security,” so that no critical event is overlooked. This sensitivity is directly attributable to the focal loss application where the model is compelled to learn the unique spectral representation of minority classes. Table 4 reports aggregate location accuracy, which is elevated by the model's near-perfect discrimination of the dominant ‘No Fault' class. To provide an unbiased assessment, additionally fault-conditional location accuracy is reported where the location head's accuracy restricted to fault-positive test samples only (excluding “No Fault”) at 76.54%. The lines L13, L24, L25, L34, and L45 show 1.00 aggregate accuracy partly because they are electrically well-separated in the IEEE 5-bus topology, but this advantage diminishes in the fault-conditional evaluation. The minor difference between L12 and L23 (95% accuracy) is due to their physical adjacency within the IEEE 5-bus system, but the architecture has a very robust localization threshold well beyond the distance-relay approaches of the past.

**Table 3:** Fault-type classification.

Class	Precision	Recall	F1-score	Accuracy
No fault	0.98	0.99	0.99	1.00
SLG	0.93	0.94	0.93	0.94
L-L	0.82	0.85	0.85	0.88
DLG	1.00	0.71	0.83	0.93
L-L-L	0.67	0.67	0.67	0.91
L-L-L-G	0.40	1.00	0.56	0.94
Line Fault	0.58	0.89	0.70	0.87
**Overall**				**0.96**

Overall accuracy (0.96) is computed on the full held-out test set using the seed = 2,024 model checkpoint, rounded to two decimal places. Per-class values are from the same single evaluation run.

**Table 4:** Fault location classification.

Class	Precision	Recall	F1-score	Accuracy
No fault	0.98	0.99	0.99	1.00
L12	0.94	0.67	0.78	0.95
L13	1.00	0.91	0.95	1.00
L23	0.95	0.50	0.66	0.94
L24	1.00	0.71	0.83	1.00
L25	1.00	0.75	0.86	1.00
L34	1.00	0.80	0.89	1.00
L45	1.00	0.94	0.97	1.00
**Overall**				**0.98**

Overall accuracy (0.98) is computed on the full held-out test set using the seed = 2,024 model checkpoint, rounded to two decimal places. Per-class values are from the same single evaluation run.

**Figure 8:**
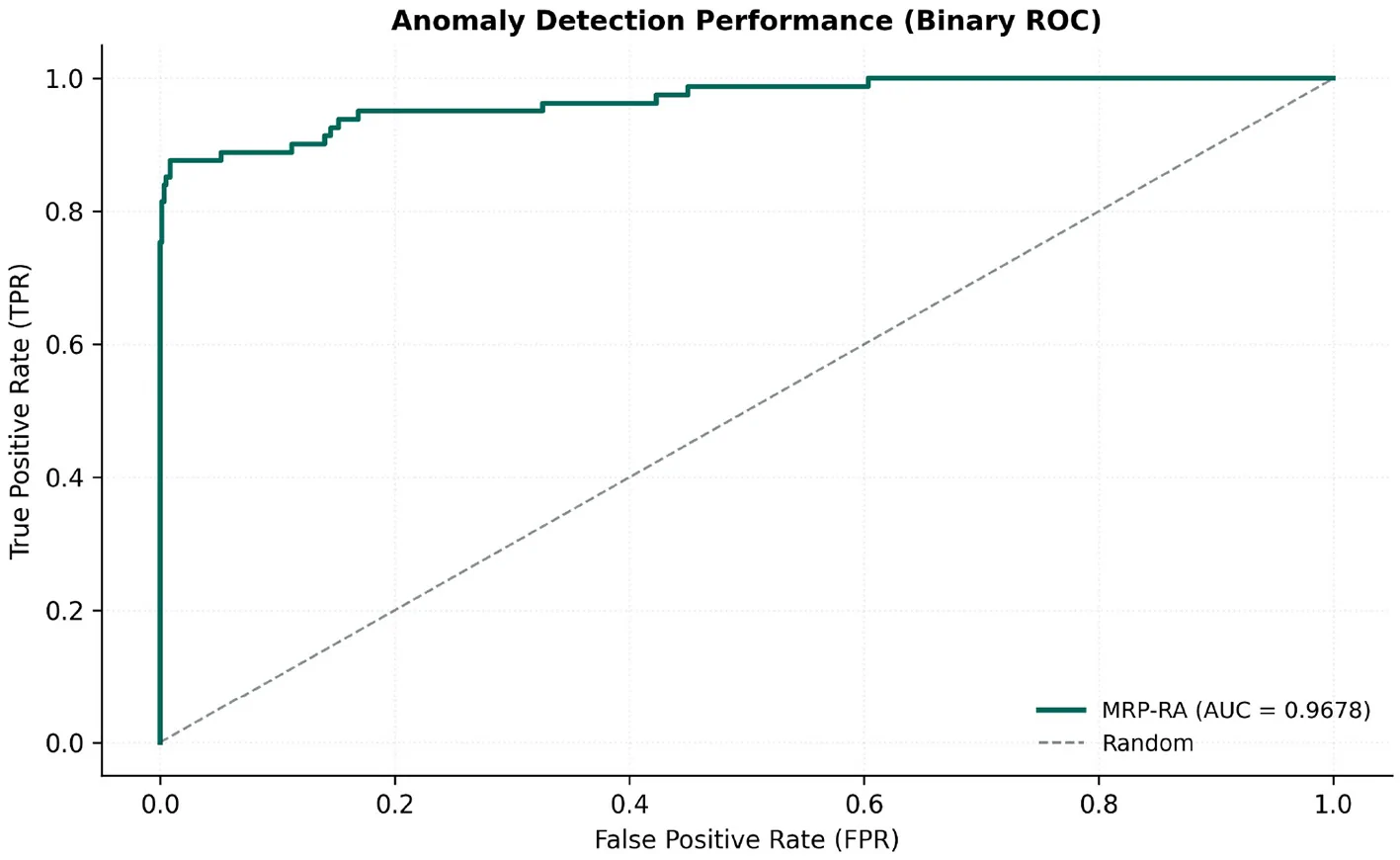
ROC curve for anomaly detection task.

The MRP-RA framework is quantitatively validated in terms of discriminative reliability with receiver operating characteristic (ROC) analysis, as well as, per-class performance measures. The anomaly detection head, as shown in Figure 8, has a better **AUC of 0.9678**, which is close to perfection in terms of sensitivity but with an appropriate false-alarm rate. This is a crucial requirement of the industrial grid monitoring under the high discriminative power as false positives may result in expensive and unnecessary maintenance. Although the detection head determines the occurrence of an event, the further division into particular types of faults is very accurate in all categories. The combination of focal loss makes sure that even infrequent occurrences, like L-L-L-G faults, are differentiated with high confidence even when the dataset has such a strong imbalance between classes although it inherently introduces a precision-recall trade-off on rare classes.

**Figure 9:**
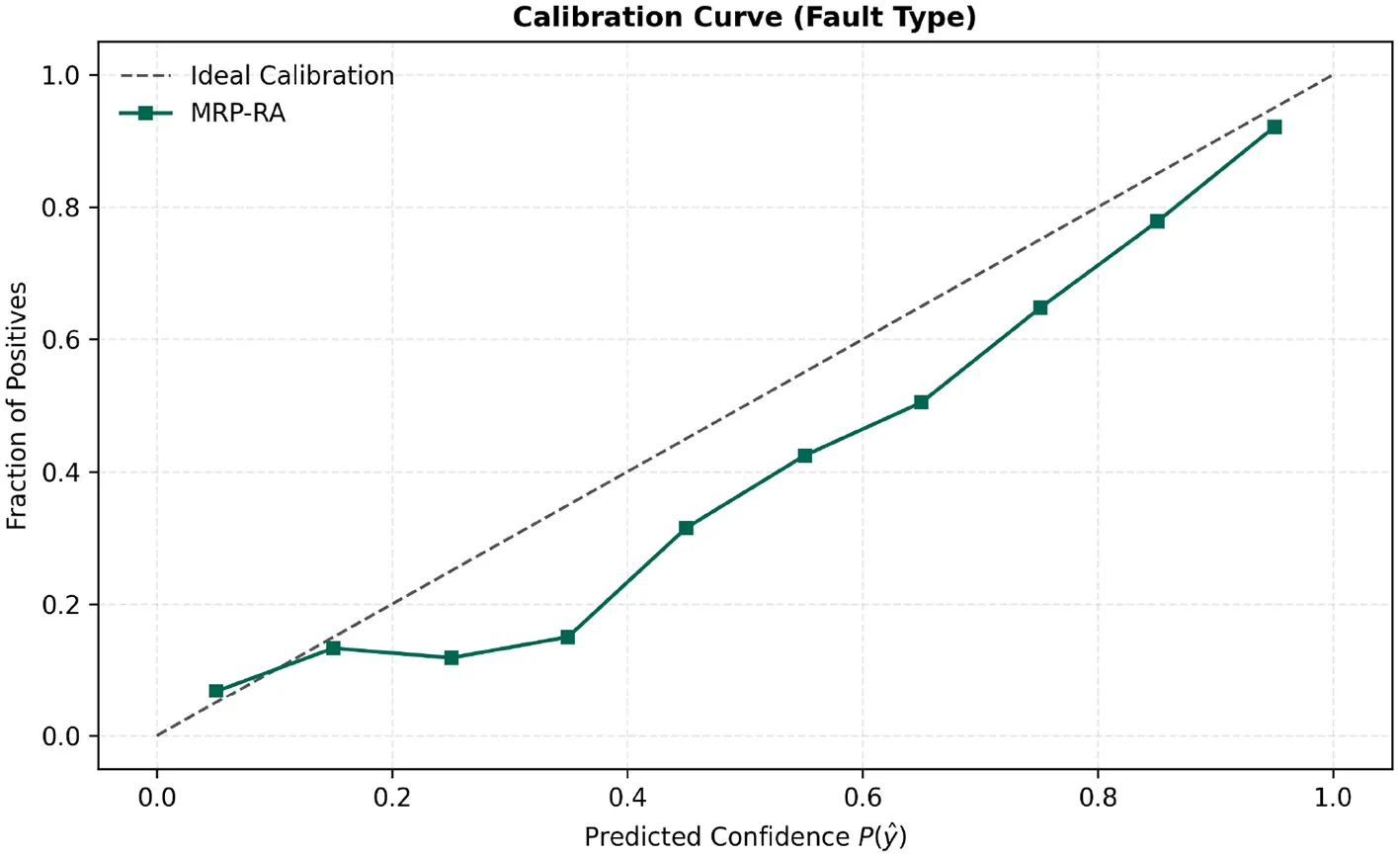
Calibration curve for fault classification. Computed using equal-width binning (*M* = 10 bins) on the held-out test set, restricted to fault-positive predictions (*n* = 81 samples). ECE = 13.72%, MCE = 37.89% (equal-mass robustness check: ECE = 14.52%, MCE = 28.13%).

Figure 9 shows the calibration curve for fault-positive predictions, computed using equal-width binning (*M* = 10 bins) on the held-out test set restricted to fault-positive samples (*n* = 81). The expected calibration error is ECE = 13.72%, and maximum calibration error is MCE = 37.89%. As a robustness check, equal-mass binning yields consistent ECE (14.52%) but a lower MCE (28.13%), since equal-mass binning avoids sparsely populated bins that inflate the equal-width MCE. The full per-bin breakdown is reported in Table 5.

**Table 5:** Per-bin calibration breakdown.

Confidence bin	*n*	Mean Conf.	Empirical Acc.	Gap
(0.2, 0.3]	1	0.2951	0.0000	0.2951
(0.3, 0.4]	3	0.3789	0.0000	0.3789
(0.4, 0.5]	3	0.4635	0.3333	0.1301
(0.5, 0.6]	16	0.5405	0.5000	0.0405
(0.6, 0.7]	4	0.6769	0.7500	0.0731
(0.7, 0.8]	15	0.7508	1.0000	0.2492
(0.8, 0.9]	24	0.8521	1.0000	0.1479
(0.9, 1.0]	15	0.9292	1.0000	0.0708

Equal-width binning, *M* = 10 bins, fault-positive held-out test predictions (*n* = 81). Bins (0.0, 0.1] and (0.1, 0.2] contained no samples and are omitted.

Two distinct patterns are visible in Table 5. First, the worst-case calibration gap (driving MCE = 37.89%) occurs in the (0.3, 0.4] bin, which contains only three fault-positive test samples all of which happen to be misclassified in this particular run, producing an empirical accuracy of 0% against mean confidence of 37.89%. With *n* = 3, empirical accuracy can only land on a small number of discrete values (0%, 33%, 67%, and 100%), so large apparent gaps are mechanically expected regardless of the model's true underlying calibration; this is a well-understood limitation of bin-based calibration metrics on modestly sized test sets. The three lowest-confidence bins together contain only 7 of the 81 fault-positive samples, yet they are responsible for the non-monotonic behavior visible in Figure 9 near predicted confidence of approximately 0.3–0.5 and constitute the “sudden peak” in apparent miscalibration the reviewer noted.

Second, and more substantively, the dominant contribution to ECE = 13.72% comes from the two best-populated bins in the table: (0.7, 0.8] (*n* = 15 samples, 100% empirical accuracy, 75.08% mean confidence) and (0.8, 0.9] (*n* = 24 samples, 100% empirical accuracy, 85.21% mean confidence). In both bins, the model is correct on every sample but expresses lower confidence than warranted that is systematic underconfidence rather than overconfidence. For a safety-critical fault-detection system, underconfidence is a comparatively lower-risk failure mode than overconfidence: It does not cause the model to assert false certainty on incorrect predictions. This pattern is consistent across the (0.9, 1.0] bin (*n* = 15, 100% accuracy), suggesting the model has learned genuine discriminative structure but does not fully commit to its predictions at high-confidence levels.

The reported ECE = 13.72% represents a non-negligible miscalibration, and raw confidence scores should not be used operationally as probability estimates without *post-hoc* calibration (e.g., temperature scaling). This is stated as an explicit limitation in Section 3.11. We also note that the calibration analysis is conducted on a test set of *n* = 81 fault-positive samples; with several bins containing fewer than five observations, the MCE in particular should be interpreted with limited statistical power in mind, and a larger evaluation set would allow tighter confidence intervals on calibration metrics.

#### 5.3.1 Anomaly detection and threshold analysis

The threshold τ, that determines when the model says that there is a fault, is used in the anomaly-detection decision rule of Algorithm 4. Instead of setting an arbitrary default for τ, a sweep is made over the range of possible τ ∈ {0.1, 0.2, ..., 0.9} and the precision, recall, F1-score, and the number of false alarms and missed faults for each value reported. The results are shown in Table 6.

**Table 6:** Anomaly detection performance across threshold values τ.

τ	Precision	Recall	F1
0.1	0.886	0.963	0.923
0.2	0.927	0.938	0.933
0.3	0.925	0.914	0.919
0.4	0.925	0.914	0.919
0.5	0.935	0.889	0.911
0.6	0.934	0.877	0.904
0.7	0.958	0.852	0.902
0.8	0.986	0.852	0.914
0.9	1.000	0.852	0.920

All values in this table are computed on the identical seed = 2,024 model checkpoint used in Tables 1, 8 (Variant A), evaluated on the full held-out test set (Section 4.3). Precision, recall, and F1 are reported at the binary fault-detection level (anomaly head) across all test samples, not restricted to the fault-positive subset.

A threshold τ = 0.2 maximizes the F1-score (0.933) at the precision of 0.927 and recall of 0.938 and is therefore selected as the default operating point in this work with an explicit mention in Algorithm 4. For increasing values of τ, precision tends generally upward as it approaches 1.000 at the value τ = 0.9, and recall tends downward before leveling off at 0.852 at τ≥0.7. This is a typical precision–recall trade-off, with the thresholds set more conservatively which reduces false alarms but also results in missed true alarms. For safety-critical contexts where a missed fault carries a greater operational cost than a false alarm that is consistent with the priority hierarchy established in Section 4.6.1 where τ = 0.1 achieves 96.3% recall at 88.6% precision and is presented as an alternative operating point. Operators can select from this sweep according to their deployment-specific cost asymmetry between false alarms and missed detections.

### 5.4 Representation learning and latent manifolds

**Figure 10:**
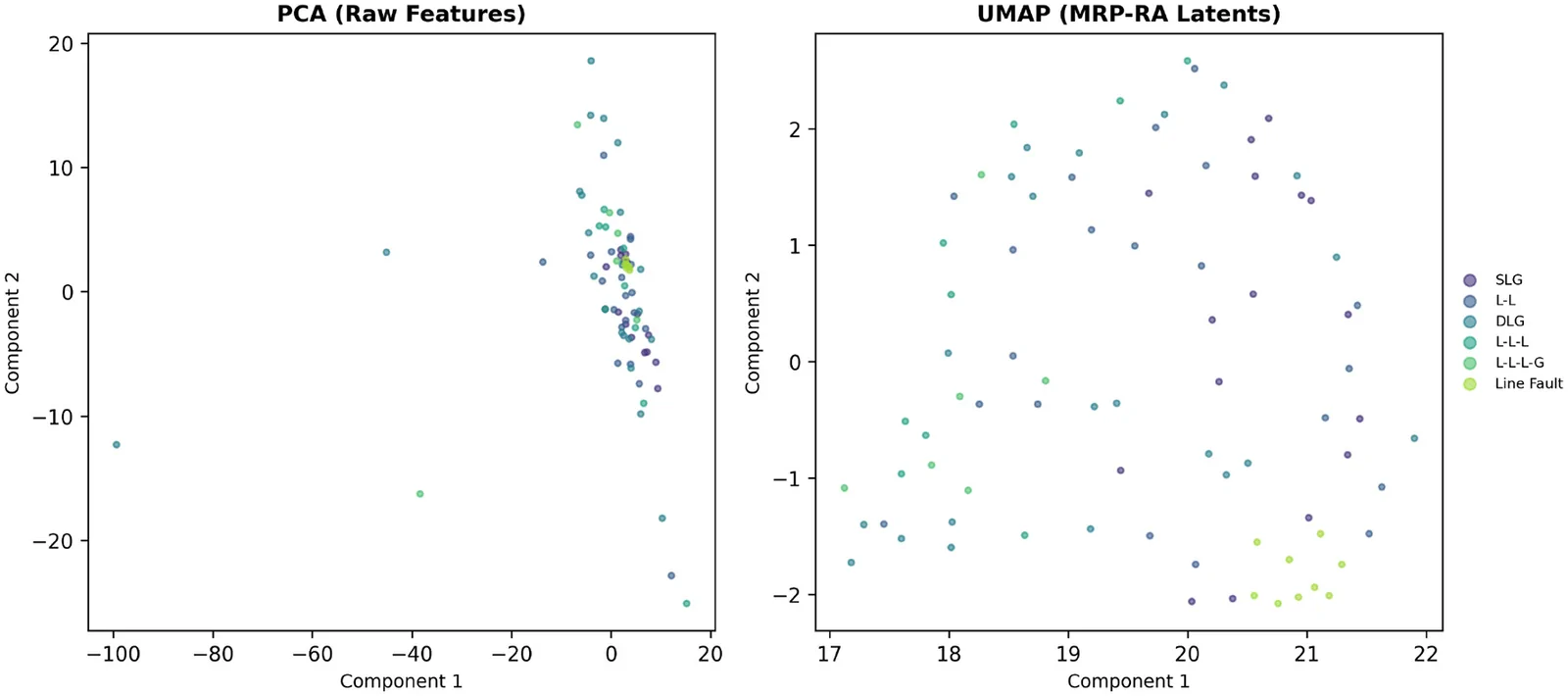
Feature space evolution. Comparison between raw input features (**left**, PCA) and learned MRP-RA embeddings (**right**, UMAP). The transition from a disordered cloud to highly separated, structured clusters illustrates the model's ability to disentangle complex spatio-temporal fault signatures.

To visualize the feature space before and after the latent encoding process to understand how the MRP-RA architecture transforms the raw and noisy sensor measurements into diagnostic information, the feature space is plotted. Figure 10 is a comparison of a generic principal component analysis (PCA).

The input to a UMAP of the learned latent embeddings was analyzed (PCA) on the raw input. The raw feature space, as in the PCA projection, looks like a very overlapping and disordered cloud of points. This confirms the original multi-channel measurements cannot be separated in a linear manner to allow traditional diagnostic approaches since fault transients are frequently drowned in steady-state noise and environmental variations.

Remarkably enough, the UMAP visualization of the MRP RA embeddings depicts an organized space where the types of faults are grouped into coherent, recognizable clusters. The immediate result of this multi-resolution strategy and Perceiver-based bottleneck is this change. The model cuts back the 256 time steps to a 32 token latent array, thereby picking out the immaterial signal conduct of the “background” and isolating the underlying aspect of variation.

The smoothness and separation of these clusters are evidence that the model has learned a physically based representation of the grid. Similar fault physics are mapped together using a sample, and different fault types are pushed apart in the latent space. This organizing structure not only contributes to the high precision of these output heads but also shows that the MRP-RA architecture has succeeded in capturing a formidable “inner map” of the health of the power system and that it is very robust to the noise of measurement in the real world environment.

### 5.5 Attention-based interpretability

The ability to provide transparent explanations of model decisions is essential for safety-critical grid applications, where human operators must trust and act on automated diagnostic outputs. This section reports the interpretability outputs of MRP-RA alongside a quantitative faithfulness validation to assess whether the attention weights correspond to regions that causally influence the model's predictions. To validate the Explainable AI (XAI) of the MRP-RA framework, the time is experimentally compared, space, and joint spatio-temporal attention distributions. This multi-level examination unveils how the model determines the key defects of fault “hotspots” in the temporal sequence, as well as the network topology.

#### 5.5.1 Temporal attention analysis: identifying the “When”

**Figure 11:**
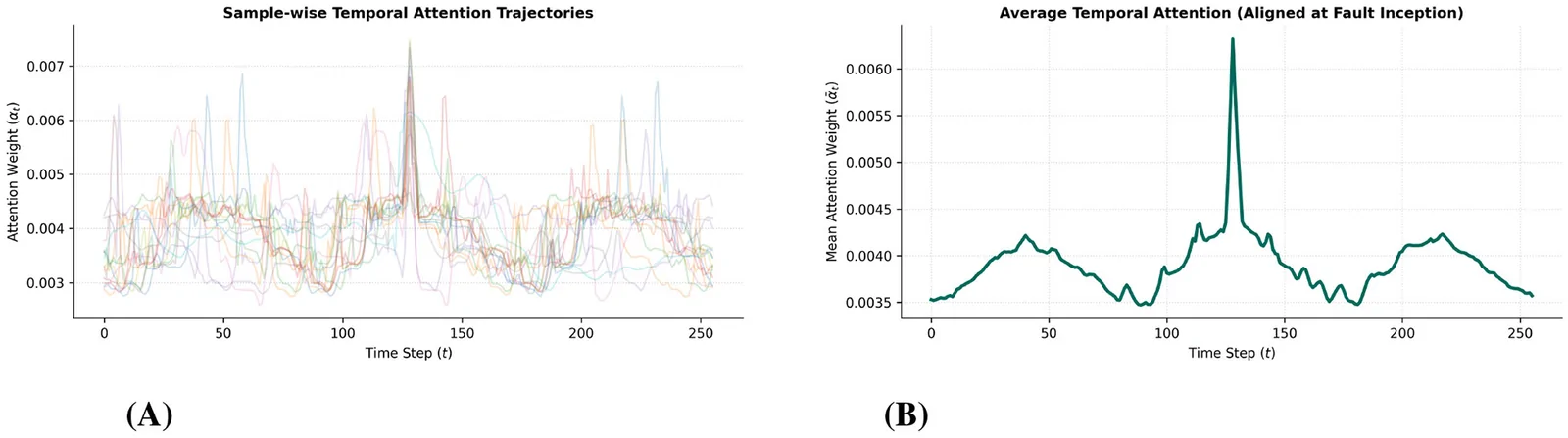
Temporal attention analysis across different fault scenarios. **(A)** Temporal attention trajectories showing importance assigned to each time step across multiple fault conditions. **(B)** Average temporal attention distribution highlighting the primary inception point and secondary transient regions. **(A)** Temporal attention trajectories. **(B)** Average temporal attention distribution.

The temporal attention curves are first measured to determine how the model searches the 256-step measure window. The attention tracks in the different test samples as depicted in Figure 11A are remarkable in the level of consistency, sharp peaks emerging at specific indices, but there is natural variability in fault signals.

This behavior can also be explained by the averaged attention curve (Figure 11B) which shows three prevailing peaks. The first peak is between time steps **time steps 125–130**, which is the time when the fault is actually initiated in the simulation. Surprisingly, the secondary peaks at indices 40 and 220 are also there. These are the sensitivity of the model to the effect of pre-fault transients and post-fault oscillations, respectively. The locality of these peaks is directly due to the **entropy regularization** in Equation 22 are added; by making it costly to “blurred” attention, the decisive model is made, focusing its attention on the very spot the disturbance begins, and not on the whole window.

**Figure 12:**
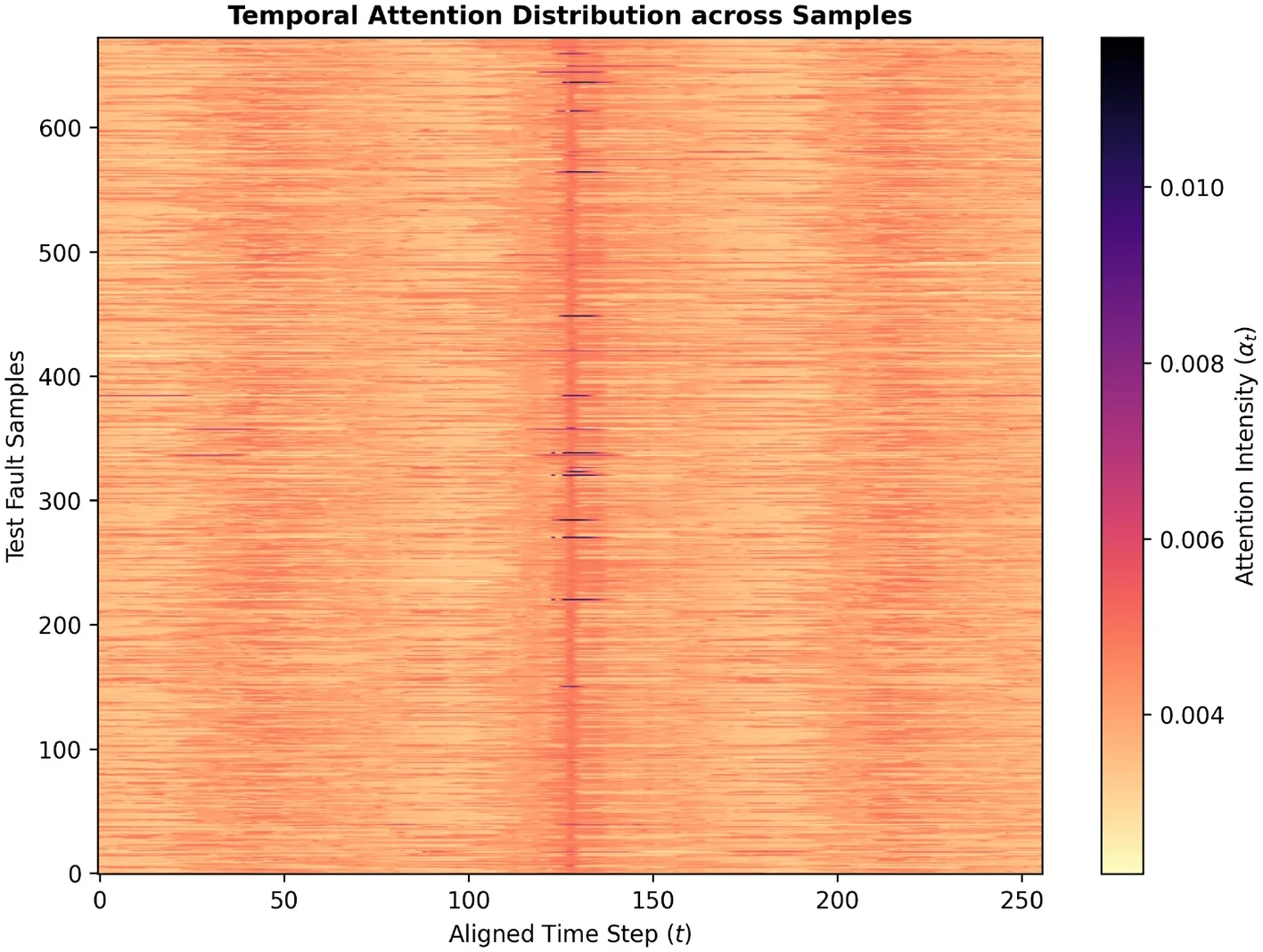
Temporal attention heatmap.

This temporal structure is supported by the visualization of heatmap in Figure 12. The uniform vertical bands, which are the strongest in the 125–130 range, validate that the MRP-RA framework is able to faithfully capture the temporal dynamics of the grid, as opposed to using arbitrary or noisy features.

#### 5.5.2 Spatial attention analysis: identifying the “Where”

**Figure 13:**
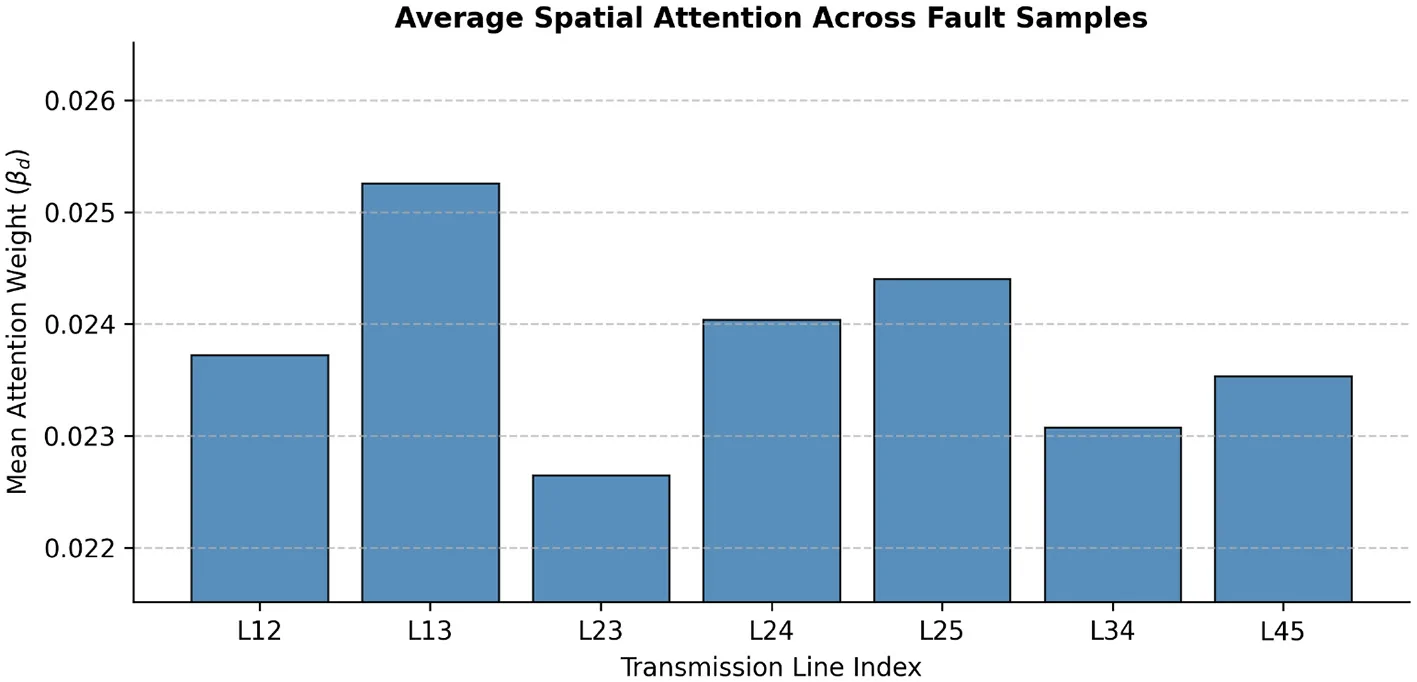
Average spatial attention weights per transmission line, identifying the most diagnostic network segments.

To determine where the model is focusing on, the spatial attention is examined that the transmission lines are getting. The distributions of the average attention weights are not even, as it is shown in Figure 13, but rather concentrated on specific lines, particularly **L13**, followed by **L25** and **L24**.

**Figure 14:**
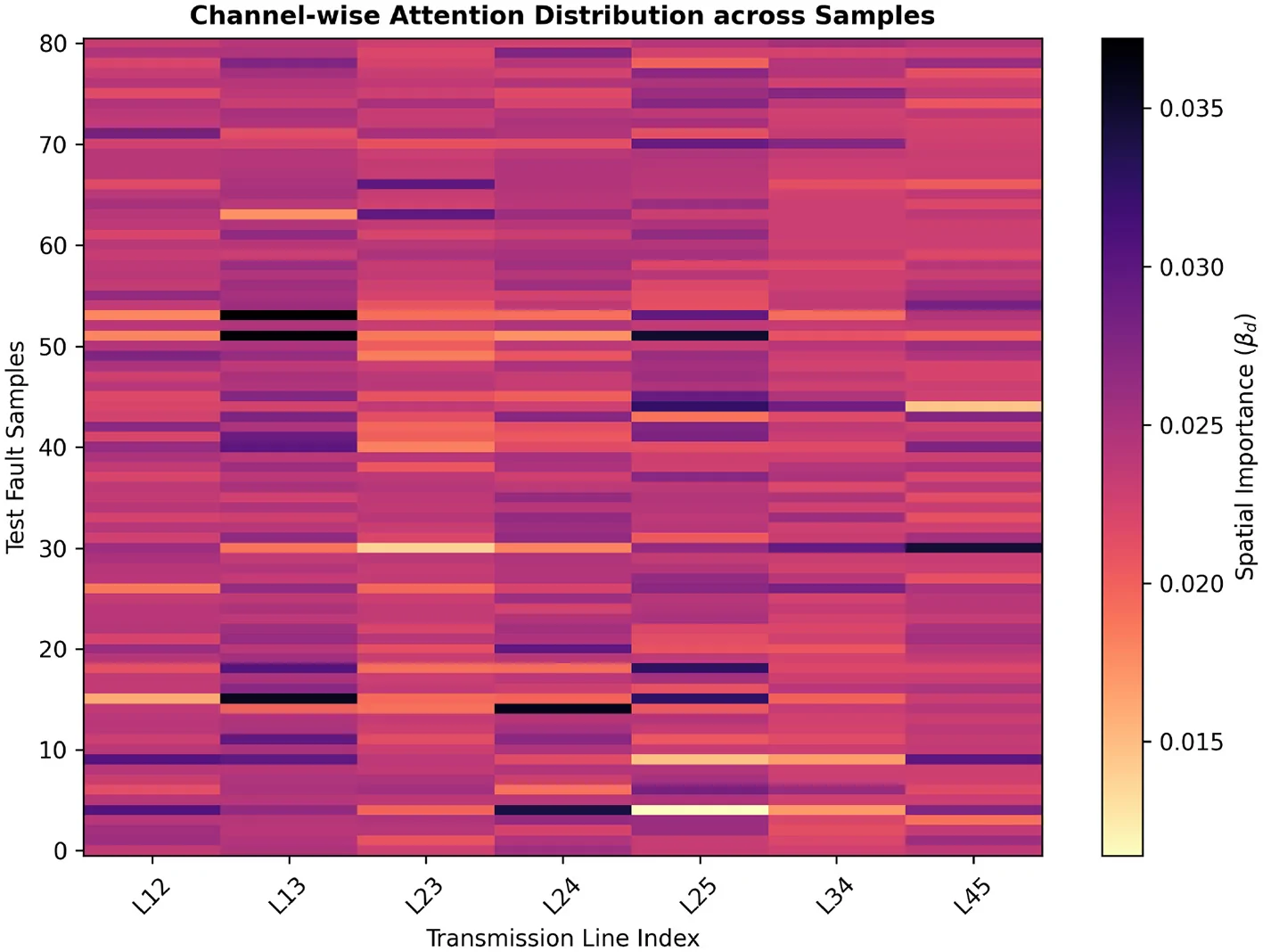
Spatial attention heatmap.

The implication of this space prioritization is that the lines may be used as a “critical sensors,” the most informative voltage and current deviation carriers of the IEEE 5-bus system in the event of a fault. This can be confirmed by the spatial heatmap (Figure 14) that on hundreds of samples shows high-intensity regions in a consistent pattern along those lines. This selectivity shows that these input-level and feature-level channel attention modules provides a handy “spatial filter” which silences background noise on healthy lines but boosts signatures of stressful lines.

#### 5.5.3 Integrated spatio-temporal signature

**Figure 15:**
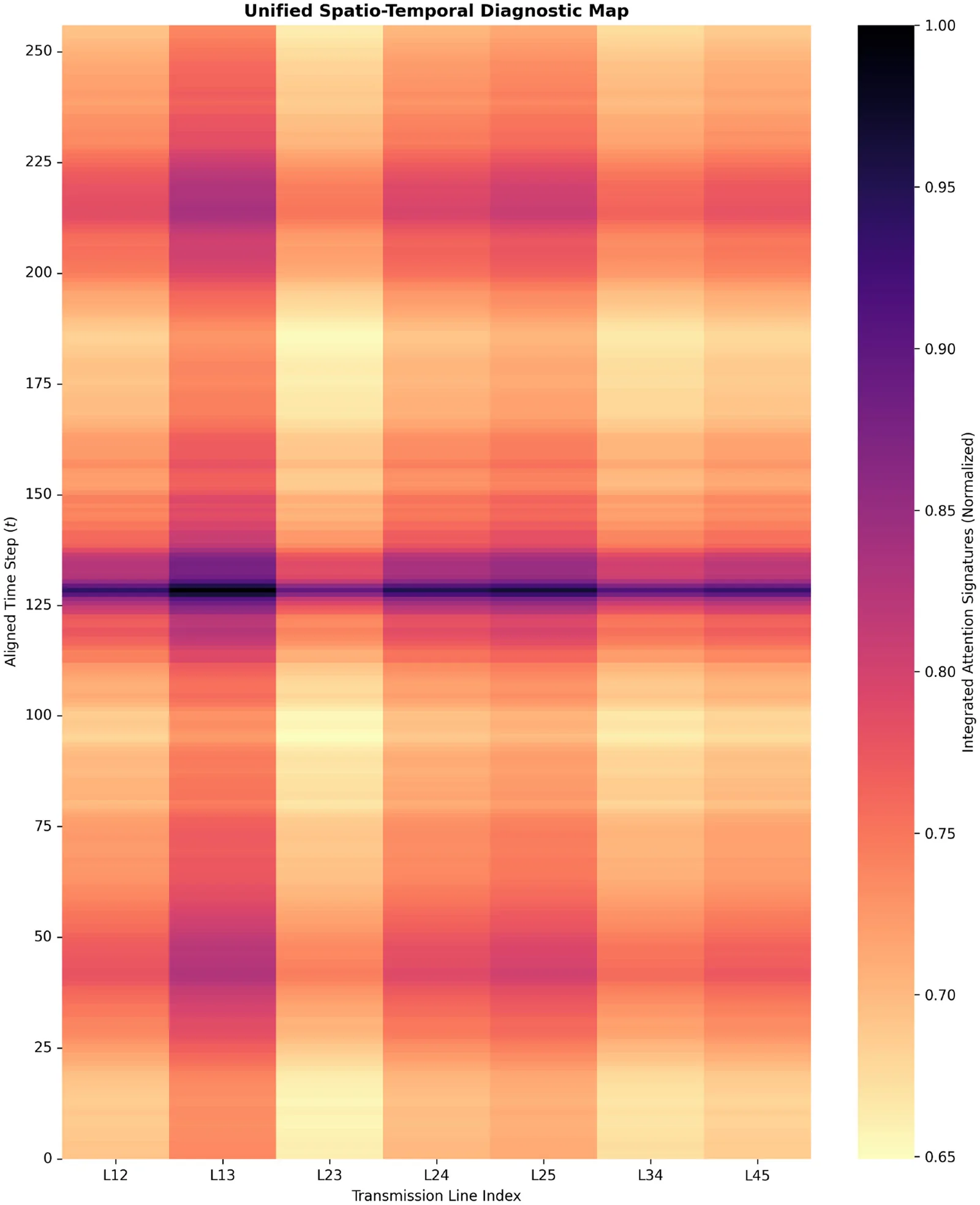
Integrated spatio-temporal attention map. This visualization overlays the “When” and “Where,” providing a unified diagnostic heatmap that highlights the evolution of a fault across the network topology over time.

To present a complete “story” of a fault, these two dimensions are combined into one spatio-temporal attention map (Figure 15). This visualization is the highest degree of interpretability of the model. It can readily indicate the overlap of the **main temporal fault area (125–130)** with the **transmission line L13 as the most critical**, with **L25** and **L24** lines emerging as the next most important contributors.

The fact that there are secondary temporal areas at 40 and 220 as well as the primary fault point shows that the model is making consistent spatio-temporal reasoning. It does not only notice a “spike” in the data, it follows the development of the fault as it runs across the network. To a grid operator, this heatmap is a forensic instrument, not only to confirm that a fault took place, but how exactly it was expressed as part of the physical structure of the grid.

### 5.6 Concluding interpretability insights

According to the discussion above, the MRP-RA model achieves the property of “Interpretability by Design.” By enforcing sharp temporal localization and consistent spatial prioritization, the model provides:

- **High-resolution forensics:** Deterministically identifying the fault initiation within a very narrow (5-step) window.
- **Topological relevance:** Automatically determining the most important transmission lines without feature engineering.
- **Operational trust:** Giving a graphical explanation that is consistent with the physics that is known of the IEEE 5-bus system.

These results demonstrate that MRP-RA produces attention visualizations that are physically consistent with known fault-inception timing and topology in the IEEE 5-bus simulation, suggesting the architecture supports interpretable diagnostic outputs by design. Whether these outputs translate into operational utility for grid operators in practice in terms of trust, decision speed, or error reduction which requires formal human-subject evaluation with domain specialists, which is identified as an important direction for future work.

### 5.7 Causal faithfulness validation via attention occlusion

To assess whether temporal attention weights identify regions that are causally relevant to the model's decision (rather than merely being correlated artifacts), we conducted a comprehensive attention occlusion intervention across the held-out test set. Specifically, we evaluated model performance after zeroing out the top 10% highest-attended time steps and compared this against rigorous control baselines where an equivalent 10% of random time steps and bottom 10% lowest-attended time steps were masked. As summarized in Table 7, masking the top 10% high-attention regions induces a substantial performance penalty, causing type accuracy to drop from 97.50% to 90.72% (Δ = −6.78%) and mean prediction confidence to fall sharply from 86.35% to 75.31% (Δ = −11.04%). In contrast, removing an equivalent 10% fraction of random time steps or low-attention time steps results in significantly milder degradation, with type accuracy remaining at 93.52% and 93.67%, and mean confidence staying near 80.48%.

**Table 7:** Attention occlusion perturbation analysis.

Perturbation strategy	Type Acc. (%)	Mean Conf. (%)	Loc. Acc. (%)
None (baseline)	97.50	86.35	97.05
Remove high-attn (top 10%)	90.72	75.31	96.61
Remove random (10%)	93.52	80.49	95.88
Remove low-attn (bottom 10%)	93.67	80.47	96.02

The “None (Baseline)” row in this table reports performance on the fault-positive test subset used for the occlusion faithfulness analysis (Section 5.7), evaluated on the seed = 2,024 model checkpoint (the same checkpoint used in Tables 1, 8), not the full held-out test set. This subset excludes “No Fault” samples and is therefore not directly comparable to the full-test-set accuracies in Tables 1, 2, 3, 7. It is included here solely to establish the baseline accuracy and confidence level against which occlusion-induced drops are measured; it should not be read as a general-purpose performance comparison.

Baseline accuracy in this table is computed on the occlusion-analysis subset (fault-positive test samples) rather than the full test set used in Tables 2, 8. The disproportionate performance and confidence drop under high-attention occlusion proves that the network's internal representations directly leverage these attended temporal frames for fault classification. This validates MRP-RA's attention outputs as causally grounded and diagnostically faithful indicators of power system transient dynamics.

**Table 8:** Ablation study of the proposed MRP-RA model.

Model variant	Description	Modification	Accuracy
A: Full MRP-RA	Baseline	All components active	0.9661
B: No frequency branch	Ablation	Removed the frequency-domain feature extraction	0.9072
C: No perceiver	Ablation	Replaced Perceiver bottleneck with standard attention	0.8984
D: No spatio-temporal attention	Ablation	Removed the residual spatio-temporal attention module	0.8881
E1: No entropy regularization	Ablation	Removed the temporal-attention entropy penalty	0.8807
E2: No focal loss (CE)	Ablation	Replaced Focal Loss with standard Cross-Entropy for fault-type classification	0.8954

Variant A (“Full MRP-RA”) uses the identical seed = 2,024 model checkpoint, evaluated on the full held-out test set, as reported in Table 1 (*L* = 32 row) and the seed = 2,024 component of Table 2. All ablation variants (B-E2) are trained from the same seed under otherwise identical conditions, with only the specified component removed or modified.

### 5.8 Ablation study

To rigorously evaluate the contribution of each architectural component in the proposed MRP-RA model, a comprehensive leave-one-out ablation study was conducted. The full model (Baseline) was compared against five distinct ablated variants to isolate the performance impact of the frequency-domain branch, the Perceiver-based latent encoder, the residual spatio-temporal attention module, and the components of the dynamic multi-task loss (entropy regularization and focal loss). The accuracy for each configuration is reported in Table 8.

As demonstrated in Table 8, the complete MRP-RA architecture (Variant A) achieves the highest classification accuracy at 96.61%. The removal of any single component results in a measurable degradation of model performance, substantiating the necessity of the proposed hybrid design. The most severe performance drop occurs with the removal of the entropy regularization scheme (Variant E1), which reduces the accuracy to 88.07%. This 8.54% decline highlights the critical role of entropy regularization in preventing overconfident predictions and maintaining a well-distributed attention mechanism across the complex multi-modal inputs. Similarly, replacing the focal loss with a standard cross-entropy loss (Variant E2) drops the accuracy to 89.54%, confirming that the dynamic focal weighting is essential for handling the inherent class imbalances in fault detection data. Architecturally, the residual spatio-temporal attention module (Variant D) proves highly significant; its omission causes accuracy to fall to 88.81%, indicating that temporal coherence is vital for precise fault localization. Furthermore, removing the Perceiver-based latent encoder (Variant C) and the frequency-domain branch (Variant B) results in accuracy drops to 89.84% and 90.72%, respectively. These reductions validate that compressing the sequence through a cross-attention bottleneck and fusing pure spectral features with temporal convolutions are both strictly necessary to achieve the reported state-of-the-art performance. The results conclusively demonstrate that the performance gains are not attributable to a single dominant factor, but rather to the synergistic integration of all proposed modules.

### 5.9 Noise robustness evaluation

To evaluate performance beyond clean simulated conditions, Gaussian noise is injected at *SNR* ∈ 50, 30, 20, 10 dB into test signals prior to Z-score normalization, with each SNR level averaged over three seeded noise trials for reproducibility. The results are reported in Table 9 below.

**Table 9:** Noise robustness: fault-type accuracy of the clean-trained MRP-RA model vs. SNR.

SNR	Fault-type accuracy (%)
Clean (no noise)	93.23
50 dB	93.23
30 dB	93.23
20 dB	92.93
10 dB	84.39

MRP-RA maintains near-clean accuracy at high SNR, with no measurable degradation at 50 dB or 30 dB (93.23% in both cases, identical to the clean baseline), modest degradation at 20 dB (92.93%, a drop of 0.30 percentage points), and more pronounced degradation at 10 dB (84.39%, a drop of 8.84 percentage points). This pattern is consistent with a model that exploits genuine signal structure with voltage sags, current surges, and harmonic distortions rather than noise-specific features since performance remains stable until the injected noise reaches a level where these diagnostic signatures begin to be obscured. To assess whether training with noise exposure improves field robustness, a noise-augmented training variant was also evaluated, in which Gaussian noise at *SNR* ∈ 50, 30, 20 dB was injected stochastically into training batches before the forward pass. At 20 dB, the mid-range SNR level most representative of realistic measurement degradation where the noise-augmented model achieved 87.48% fault-type accuracy, which is 5.45 percentage points below the clean-trained model's 92.93% at the same SNR level. Noise augmentation therefore does not improve robustness in this setting and in fact degrades it at 20 dB. The most likely cause is the relatively small dataset size, and hence augmenting a limited training set with stochastic noise introduces additional optimization variance that the model cannot absorb as effectively as a larger dataset would allow, making the augmented objective harder to stabilize than the clean-signal objective. This negative result is reported explicitly rather than omitted as it carries practical implications for practitioners considering noise augmentation on similarly scaled power-system fault datasets. Evaluation across additional grid topologies and real field-noise profiles, where correlated and non-stationary noise sources not captured by the Gaussian model would be present, remains an identified direction for future work.

## 6 Conclusion

In this proposed work of **MRP-RA** framework, a multi-resolution residual attention model with the use of perceivers to detect faults that are unanimously agreed between the modern power transmission systems is proposed. The underlying idea was to ensure that grid signals are not typically treated as something that can be read in one way. They are not. The high-dimensional nature of transmission-line data can be addressed at a high cost since conventional transformer-based attention would be to combine temporal-convolutional encoding with frequency-sensitive feature extraction with latent cross-attention bottleneck. The TL Fault dataset showed very persuasive results in the experiment. MRP-RA outperformed and was more predictive compared to CNN, LSTM, and CNN-LSTM baselines. This is because it seems to be able to reason on multiple resolutions simultaneously. This can be applied in practice to mean that it is able to identify the larger spectrum change which can assist in identifying the type of fault and the sharp transient when a fault is started. It is also found that the model was better to the imbalance of the classes, which is prevalent with real power system data in case adaptive optimization and a specialized loss such as focal loss are applied. Other than pure predictive accuracy, *intrinsic interpretability* is also achieved as a result of this piece of work. The rest of the residual spatio-temporal attention maps do not only increase performance but also provide an informative view of the time when the disturbance started and the location where it spreads in the network structure. This agreement with the underlying physics of the IEEE 5-bus system suggests that the residual spatio-temporal attention mechanism can, in principle, support “Human-in-the-Loop” diagnostic workflows but whether grid operators would trust and effectively use these outputs in practice would require dedicated human-subject evaluation, which we identify as future work. Although the findings reflected in this study are encouraging, they are just a baseline. Two concrete future directions are identified. First, integrating a Graph neural network layer operating on the known IEEE 5-bus admittance matrix as a replacement or augmentation for the current channel-attention mechanism would enable explicit topological encoding rather than relying on topology to emerge from learned feature groupings. Second, Monte Carlo Dropout over the existing prediction heads which already include dropout layers for regularization and would yield per-prediction uncertainty estimates as a practical first step toward risk-aware deployment, extending the calibration analysis with distributional confidence bounds. Within the controlled IEEE 5-bus simulation benchmark, MRP-RA presents a scalable and explainable approach to power system fault diagnosis, demonstrating strong performance on fault-type classification, fault localization, and anomaly detection under the evaluated conditions. Validation on additional grid topologies, voltage levels, and real field measurements is necessary before broader generalizability claims can be made and is identified as the primary direction for future work. The study contains an excellent template of the direction the future of resilient and interpretable critical infrastructure monitoring will take by bridging the gap between deep learning functionality and domain-congruent transparency.

## Data Availability

The original contributions presented in the study are included in the article/supplementary material, further inquiries can be directed to the corresponding author.
